# Aquaporins in Acute Brain Injury: Insights from Clinical and Experimental Studies

**DOI:** 10.3390/biomedicines13061406

**Published:** 2025-06-07

**Authors:** Stelios Kokkoris, Charikleia S. Vrettou, Nikolaos S. Lotsios, Vasileios Issaris, Chrysi Keskinidou, Kostas A. Papavassiliou, Athanasios G. Papavassiliou, Anastasia Kotanidou, Ioanna Dimopoulou, Alice G. Vassiliou

**Affiliations:** 1 First Department of Critical Care Medicine, School of Medicine, National and Kapodistrian University of Athens, Evangelismos Hospital, 10676 Athens, Greece; skokkoris2003@yahoo.gr (S.K.); vrettou@hotmail.com (C.S.V.); n.lotsios96@gmail.com (N.S.L.); vasilisiss@gmail.com (V.I.); chrysakes29@gmail.com (C.K.); akotanid@med.uoa.gr (A.K.); idimo@otenet.gr (I.D.); 2 First University Department of Respiratory Medicine, ‘Sotiria’ Chest Hospital, School of Medicine, National and Kapodistrian University of Athens, 11527 Athens, Greece; konpapav@med.uoa.gr; 3 Department of Biological Chemistry, School of Medicine, National and Kapodistrian University of Athens, 11527 Athens, Greece; papavas@med.uoa.gr

**Keywords:** aquaporin, traumatic brain injury, ischemic stroke, SAH, ICH

## Abstract

Aquaporins (AQPs) are a family of transmembrane water channel proteins facilitating the transport of water and, in some cases, small solutes such as glycerol, lactate, and urea. In the central nervous system (CNS), several aquaporins play crucial roles in maintaining water homeostasis, modulating cerebrospinal fluid (CSF) circulation, regulating energy metabolism, and facilitating neuroprotection under pathological conditions. Among them, AQP2, AQP4, AQP9, and AQP11 have been implicated in traumatic and non-traumatic brain injuries. The most abundant aquaporin (AQP) in the brain, AQP4, is essential for fluid regulation, facilitating water transport across the blood–brain barrier and glymphatic clearance. AQP2 is primarily known for its function in the kidneys, but it is also expressed in brain regions related to vasopressin signaling and CSF dynamics. AQP9 acts as a channel for glycerol and lactate, thus playing a role in metabolic adaptation during brain injury. AQP11, an intracellular aquaporin, is involved in oxidative stress responses and cellular homeostasis, with emerging evidence suggesting its role in neuroprotection. Aquaporins play a dual role in brain injury; while they help maintain homeostasis, their dysregulation can exacerbate cerebral edema, metabolic dysfunction, and inflammation. In traumatic brain injury (TBI), aquaporins regulate the formation and resolution of cerebral edema. In non-traumatic brain injuries, including ischemic stroke, aneurysmal subarachnoid hemorrhage (aSAH), and intracerebral hemorrhage (ICH), aquaporins influence fluid balance, energy metabolism, and oxidative stress responses. Understanding the specific roles of AQP2, AQP4, AQP9, and AQP11 in these brain injuries may lead to new therapeutic strategies to mitigate secondary damage and improve neurological outcomes. This review explores the function of the above aquaporins in both traumatic and non-traumatic brain injuries, highlighting their potential and limitations as therapeutic targets for neuroprotection and recovery.

## 1. Introduction

Brain injuries are entities commonly encountered in the intensive care unit (ICU) and can be classified into two main categories: traumatic brain injury (TBI) and non-traumatic brain injuries (NTBIs).

Traumatic brain injury (TBI) results from an external mechanical force, such as a blow to the head, leading to primary and secondary injury mechanisms. The primary injury occurs at the moment of impact, causing direct neuronal and vascular damage, while the secondary injury involves delayed biochemical and cellular responses, including inflammation, oxidative stress, and edema formation [1,2].

Non-traumatic brain injuries (NTBI) result from internal pathological processes such as stroke, aneurysmal subarachnoid hemorrhage (aSAH), and intracerebral hemorrhage (ICH). The role of aquaporins under these conditions has increasingly been recognized. In acute ischemic stroke (AIS), cerebral blood flow is disrupted, leading to energy failure, ionic imbalance, and water influx into astrocytes, causing cytotoxic edema [3]. Aneurysmal subarachnoid hemorrhage occurs due to the rupture of an intracranial aneurysm, leading to blood accumulation in the subarachnoid space. This condition triggers a cascade of secondary injuries. Within the first 72 h, early brain injury (EBI) occurs, resulting in blood–brain barrier (BBB) disruption, inflammation, and cerebral edema. In the delayed cerebral ischemia (DCI), vasospasm, microvascular dysfunction, metabolic stress, and impaired cerebrospinal fluid (CSF) clearance can result in secondary ischemia [4,5]. Intracerebral hemorrhage is a life-threatening condition characterized by bleeding within the brain parenchyma and is often caused by hypertension, vascular malformations, or anticoagulant therapy. ICH-induced brain injury involves hematoma expansion, leading to mechanical tissue damage and increased intracranial pressure (ICP). Perihematomal edema (PHE), which is the swelling that occurs around a hemorrhage, inflammation, and secondary injuries, such as oxidative stress and immune system activation, contribute to delayed neuronal damage [6].

A common feature among these conditions is the formation of cerebral edema and the disruption of the BBB, which exacerbate neurological damage and complicate recovery. Aquaporins (AQPs) are a family of integral membrane proteins that function as selective water channels, facilitating the movement of water molecules across cell membranes [7,8,9]. These proteins are essential for maintaining water homeostasis in various tissues, including the central nervous system (CNS) [10].

In this review, we present and discuss the findings from clinical and experimental studies on AQP4, AQP2, AQP9, and AQP11 in acute brain injuries. The putative mechanisms of their involvement in TBI and NTBI, and the perspectives and limitations of their use as therapeutic targets are also be discussed. The purpose is to integrate experimental evidence and clinical insights of AQPs in acute brain injuries, with a focus on their roles in pathophysiology, therapeutic potential, and translational implications.

## 2. Expression and Function of Aquaporins in the Brain

Among the 13 known mammalian aquaporins, AQP4 is the most highly expressed in the brain and is predominantly localized to the perivascular end-feet of astrocytes, ependymal cells, and glial limiting membranes. It is involved in water movement, edema formation, and the clearance of interstitial solutes. It is found at the BBB and brain–CSF interfaces, where it plays a crucial role in regulating water movement between the brain parenchyma, blood vessels, and the glymphatic system [11,12,13,14,15]. It also contributes to potassium homeostasis by working in tandem with astrocytic potassium channels, thereby influencing neuronal excitability and synaptic plasticity [16]. AQP2, AQP9, and AQP11 are also present in the brain, although with more specialized roles [10]. AQP9 is expressed in astrocytes and some neurons and transports lactate, glycerol, and other solutes [17,18]. It is linked to metabolic regulation under stress or injury [19,20]. AQP2 is expressed primarily in the kidney (collecting ducts), where it plays a key role in vasopressin-regulated water reabsorption [21]. It shows minimal expression in the brain under normal conditions [22], where it seems to have an indirect role via systemic water balance [23]. Finally, AQP11 is an intracellular aquaporin that is localized to the endoplasmic reticulum (ER). It is expressed in various tissues, including the brain, particularly in astrocytes and glial cells. Its function is not fully understood, but it seems to be involved in ER homeostasis, oxidative stress regulation, and cell survival under stress conditions [24].

**Figure 1:**
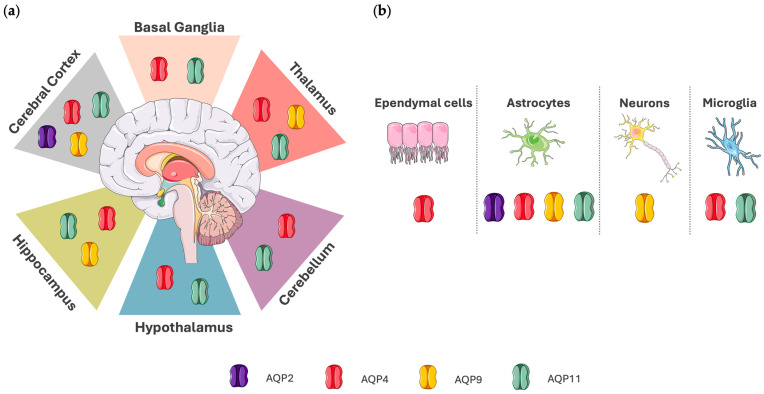
Depiction of aquaporin expression in the human brain. (**a**) Localization of AQP expression in specific brain regions. AQP2 is found mainly in the cerebral cortex. AQP4 and AQP11 are localized in the basal ganglia, cerebellum, cerebral cortex, hippocampus, hypothalamus, and thalamus. AQP9 is expressed in the cerebral cortex, hippocampus, and thalamus. (**b**) Aquaporin expression in cell types of the central nervous system (CNS). AQP2 is expressed in astrocytes. AQP4 is expressed in astrocytes, ependymal cells, and microglia. AQP9 is expressed in astrocytes and neurons, whereas AQP11 is localized in astrocytes and microglia. Data regarding AQP localization in the human brain were acquired from The Human Protein Atlas (proteinatlas.org), which was accessed on 4 April 2025. Aspects of this figure were adapted with permission from the Servier Medical Art library, which is available under the Creative Commons license.

Figure 1 shows the localization of AQP2, AQP4, AQP9, and AQP11 in the human brain, and Table 1 summarizes their roles.

## 3. Clinical Studies of Aquaporins in Acute Brain Injury

In the context of acute brain injury, the dysregulation of AQPs has been increasingly linked to the development of cerebral edema, neuroinflammation, and secondary injury progression. In addition to AQP4, clinical studies also implicate AQP2 and AQP9 in injury-specific fluid dynamics, inflammatory signaling, and systemic responses. Altered expression patterns of AQPs, identified through cerebrospinal fluid, serum biomarkers, histological samples, and genetic studies, have revealed their potential as both prognostic markers and therapeutic targets. Clinical evidence also implicates AQP2 and AQP9 in pathophysiological processes beyond mere water transport. These include systemic fluid imbalance, inflammation, and metabolic regulation. Their involvement appears to be context-dependent and influenced by injury type, timing, and systemic responses. In this section, we present and contextualize clinical findings related to aquaporin expression and modulation across various acute brain injury types, with a focus on their potential as biomarkers or therapeutic targets.

### 3.1. Aquaporins in Traumatic Brain Injury

Traumatic brain injury remains a leading cause of death and long-term disability worldwide. Central to its pathophysiology are cerebral edema and neuroinflammation, both of which contribute to secondary brain damage and cognitive decline. AQPs are increasingly recognized as key molecular mediators in these processes. In addition to AQP4, other isoforms, including AQP2 and AQP9, also play important roles in modulating injury outcomes.

Post-mortem and clinical studies have consistently reported altered AQP4 expression following TBI. In mild cases, plasma neuron-derived exosomes (NDEs) show a nearly 9-fold increase in AQP4 levels acutely and a 3.6-fold increase chronically compared with those in controls [45]. Similarly, elevated cerebrospinal fluid AQP4 levels have been observed in severe TBI, linking AQP4 to brain water metabolism and edema formation [40]. Histological analyses further revealed the progressive upregulation of AQP4 between 7- and 30-days post-injury, which coincided with increased neuroinflammation and hypoxia [46].

AQP4 upregulation is often localized to astrocytes surrounding edematous tissue and is frequently associated with vascular endothelial growth factor (VEGF) activity [47]. In diffuse axonal injury, imaging studies have demonstrated a strong correlation between edema severity, axonal damage, and elevated AQP4 expression [48]. In addition to its role in water transport, AQP4 contributes to neuroinflammatory signaling. Toll-like receptor 4 (TLR4) activation in microglia induces interleukin (IL)-6 release, which, in turn, stimulates AQP4 expression in astrocytes, sustaining edema and inflammation [49].

In a study examining the long-term effects of TBI, AQP4 levels increased significantly within the first week after TBI and remained elevated, although at lower levels, for up to a year. However, there were no significant differences in AQP4 levels between those with and without cognitive impairment (CI), suggesting that its role is more prominent in the early response to TBI than in long-term cognitive outcomes [50].

Studies in blast-related TBI show laminar alterations in AQP4 expression and increased MRI-visible perivascular spaces, indicating glymphatic dysfunction and a heightened risk of neurodegeneration [51].

In addition to its functional regulation, AQP4 is also subject to genetic and molecular control. The microRNA (miR)-211-5p/MMP9/AQP4 axis is a key regulatory pathway, with reduced miR-211-5p leading to increased AQP4 expression, suggesting the potential for targeted intervention [52]. Additionally, specific *AQP4* gene polymorphisms are associated with worse clinical outcomes in TBI [53], although some studies report no variation in exon 4, highlighting the need for further genetic investigation [54].

Other aquaporins also contribute to TBI pathology. Although AQP2 is involved primarily in renal water handling, it has potential as a peripheral biomarker. Its serum levels correlate with hematoma volume in TBI patients, indicating a systemic fluid regulatory response [22].

Although less studied, AQP9 facilitates the transport of lactate and glycerol and is expressed in immune and glial cells. It may contribute to metabolic adaptation and inflammation following TBI [22].

Taken together, these clinical data consistently point to AQP4 as a central mediator of cerebral edema in TBI, with expression changes tightly linked to inflammatory signaling (e.g., IL-6, TLR4), hypoxia, and axonal damage. *AQP4* gene polymorphisms and circulating microparticles suggest their utility as both a biomarkers and possible therapeutic targets. In contrast, AQP2 appears to reflect systemic neuroendocrine responses to injury (e.g., syndrome of inappropriate antidiuretic hormone—SIADH), and AQP9 may contribute to cellular adaptation via lactate and glycerol transport. This interplay highlights the multifactorial nature of post-traumatic edema and inflammation.

Given its central role, AQP4 is being explored as a biomarker and therapeutic target. Circulating AQP4-containing microparticles in the blood may serve as non-invasive indicators of brain injury [55]. Additionally, longitudinal tracking of exosomal AQP4 could help identify individuals at risk of TBI-related neurodegeneration [45]. AQP4 dysregulation may influence cognitive outcomes by affecting water balance and glymphatic clearance in memory-relevant regions such as the hippocampus. Disruption of AQP4 polarity and expression in perivascular astrocytes has been linked to impaired waste clearance and tau accumulation, which may contribute to long-term neurodegenerative processes and post-TBI cognitive decline [45,51,56,57,58]. Pharmacologic strategies such as TLR4 inhibition have shown promise in preclinical models to reduce AQP4-mediated edema and inflammation [49].

### 3.2. Aquaporins in Acute Ischemic Stroke

Acute ischemic stroke remains a leading cause of morbidity and mortality worldwide. Cerebral edema is central to its pathophysiology, and the key role of AQPs in fluid regulation may contribute significantly to its pathophysiology.

Post-mortem studies further highlight region-specific changes in AQP4 expression in AIS. While cortical levels of AQP4 remained unchanged, white matter showed increased perivascular and plasmalemmal AQP4, corresponding to significantly greater swelling, approximately 40% in white matter versus 9% in the cortex [59]. Immunohistochemical analyses revealed that AQP4 immunoreactivity (IR) was stronger at the periphery of ischemic lesions and extended along astrocytic processes in contact with capillaries. AQP4 IR was also enhanced near subpial and subependymal surfaces, underscoring its role in edema development through vascular and surface pathways [41].

AQP4 has also emerged as a potential biomarker for prognosis. A pilot study of AIS patients treated with intravenous thrombolysis (t-PA) revealed that baseline serum AQP4 levels were inversely correlated with infarct size and neurological severity at admission, as measured by the National Institutes of Health Stroke Scale (NIHSS). Higher AQP4 levels were associated with greater neurological improvement and better outcomes at 48 h and hospital discharge [60]. Systolic blood pressure (SBP) within the first 24 h post-thrombolysis is a known predictor of neurological deterioration. AQP4 levels measured at 24 h also independently predicted clinical decline and were positively correlated with SBP, suggesting that elevated AQP4 may mediate blood pressure-induced BBB disruption via oxidative stress pathways [61].

Genetic studies provide additional insights. The *AQP4* single nucleotide polymorphism (SNP) rs9951307 has been specifically associated with increased cerebral edema after middle cerebral artery occlusion [62]. In hypertensive patients, certain *AQP9* SNPs have been linked to increased stroke risk, implicating aquaporin-related lipid metabolism in cerebrovascular vulnerability [63].

Systemic AQP activity also appears relevant. Stroke patients with hyponatremia present elevated urinary AQP2 and plasma vasopressin levels despite reduced osmolality, suggesting non-osmotic AQP2 regulation and possible kidney-brain fluid imbalance [64].

In summary, AQP4 expression exhibits dynamic and region-specific regulation, particularly in white matter, where its upregulation contributes to pronounced edema. Early elevations in serum AQP4 have shown promise as prognostic indicators, particularly in thrombolysis-treated patients. The observed associations between *AQP4* polymorphisms and stroke outcomes further underscore its potential clinical relevance. Although less extensively studied, AQP2 and AQP9 may reflect renal–brain crosstalk and cerebrovascular metabolic stress, respectively. These findings point to the value of aquaporins not only as mediators of edema but also as integrators of systemic and neural injury responses. Finally, region-specific changes in AQP4, particularly in white matter tracts and cortical networks, may also influence post-stroke recovery trajectories. Edema-related damage in motor and sensorimotor pathways, coupled with impaired astrocyte-mediated fluid regulation, could play a role in delayed neurological recovery and persistent functional deficits [59,65,66].

### 3.3. Aquaporins in Aneurysmal Subarachnoid Hemorrhage and Intracerebral Hemorrhage

Aneurysmal subarachnoid hemorrhage and ICH are severe forms of intracranial bleeding associated with high morbidity and mortality. Brain edema and secondary inflammation are major contributors to poor outcomes. A study of AQP expression in SAH demonstrated significant upregulation of AQP4 in astrocytic processes. However, their normal polarization at astrocytic end-feet was disrupted, and no expression was observed in neurons. This redistribution may impair water clearance, contributing to edema development and delayed resolution [42].

In ICH, AQP2 has shown potential as a systemic biomarker. A study comparing 33 ICH patients with healthy controls reported significantly lower serum AQP2 levels in the ICH group. Lower levels were associated with worse 90-day outcomes, although not with initial stroke severity. Sex and neutrophil count were also correlated with AQP2 concentrations, suggesting its role as an early inflammatory marker and possible indicator of renal or systemic dysregulation [21].

The expression of AQP4 in hemorrhagic brain injury is notably altered, with polarization loss at astrocytic end-feet possibly impairing glymphatic clearance and contributing to prolonged edema and neuroinflammation. AQP2 has emerged as a potential systemic biomarker with prognostic value in ICH, reflecting not only fluid dysregulation but also inflammatory activation. While data on AQP9 are limited, its role in metabolic adaptation may also be relevant in the hemorrhagic context. Collectively, these observations suggest a complex spatial and temporal pattern of aquaporin dysregulation shaped by both central and systemic injury dynamics.

The clinical findings outlined above reveal both the promise and complexity of targeting aquaporins in acute brain injury. Although the present review is organized around clinical entities commonly encountered in critical care, shared mechanistic patterns emerge across different types of acute brain injury. For example, AQP4 is consistently implicated in the regulation of cerebral edema, particularly through its polarized expression in astrocytic end-feet at the BBB and CSF interfaces. AQP2 may influence systemic water balance and intracranial pressure indirectly, especially in patients with neuroendocrine disturbances such as SIADH. AQP9 appears to support metabolic adaptation by transporting lactate and glycerol, which may, in turn, modulate inflammation and neuronal survival. Finally, AQP11, which is still under investigation, is associated with the regulation of intracellular oxidative stress and endoplasmic reticulum homeostasis.

These intersecting roles in edema formation and resolution, metabolic stress, and inflammation provide a conceptual bridge between clinical observations and the experimental findings discussed in the following section. For example, the dual role of AQP4 in both promoting edema formation and facilitating its resolution underscores the importance of understanding its temporal dynamics. While early AQP4 upregulation may exacerbate cytotoxic and vasogenic edema, later phases involve its redistribution and potential role in edema clearance via the glymphatic system. This time-dependent behavior highlights the need for therapeutic strategies that are precisely timed to either inhibit or enhance AQP4 function according to the phase of injury [11,56,59,67].

Table 2 summarizes the clinical studies and their findings involving AQP2, AQP4, AQP9, and AQP11 in acute brain injuries.

Several of the functional consequences of AQP dysregulation, particularly those involving cognitive decline after TBI and motor recovery following stroke, are thought to arise from aquaporin-related changes in neural substrates such as the hippocampus and sensorimotor cortex. Disruptions in water homeostasis, glymphatic clearance, and astrocyte polarity within these regions may impair memory processing, synaptic plasticity, and circuit-level recovery. These circuit-specific implications are further elucidated in the experimental models discussed below [48,57,59,67].

## 4. Experimental Studies of Aquaporins in Acute Brain Injury

### 4.1. AQP4

#### 4.1.1. AQP4 in Traumatic Brain Injury

As in clinical studies, experimental data also support the notion that AQP4 plays a complex and at times contradictory role in brain edema following TBI [72]. Some studies report downregulation of AQP4 after injury, impairing water clearance and worsening edema [73,74], whereas others show early upregulation linked to increased edema severity [75,76]. Loss of AQP4 polarization at astrocyte end-feet is also associated with cytotoxic edema [77,78]. Genetic or siRNA-mediated *AQP4* knockdown improves outcomes by reducing edema and injury volume [79,80,81]. AQP4 regulation appears to be region- and context-dependent and influenced by factors such as secondary insults, inflammation, and oxidative stress [82,83,84].

Table 3 extensively lists the studies on AQP4 expression in experimental TBI models.

##### Methodological Differences of Experimental Studies on the Role of AQP4 in TBI

Experimental studies on AQP4 in TBI have shown divergent findings, largely due to methodological differences. Species varied; rats were most common, but mice and sheep were also used, introducing interspecies variability. Injury models differ; most have used controlled cortical impact (CCI), whereas others have employed penetrating, closed-skull, or fluid percussion models, each of which affects the brain differently. For example, CCI models tend to show early AQP4 upregulation and redistribution associated with cytotoxic edema, whereas fluid percussion injury, which induces more diffuse damage, may result in delayed or region-specific AQP4 expression linked to vasogenic mechanisms. The timing of measurements ranged from acute (1–24 h) to subacute or delayed (up to 7 days), influencing the observed AQP4 expression and localization. Techniques including mRNA/protein quantification, immunohistochemistry, and imaging also vary. Some studies have incorporated genetic or pharmacologic interventions (e.g., *AQP4* knockouts, siRNAs, pathway inhibitors), which, while mechanistically informative, reduce comparability. Finally, studies have focused on different brain regions (e.g., the hippocampus and cortex), which differ in their susceptibility to injury. These methodological differences, such as model type, species, timing, techniques, and anatomical focus, contribute to the inconsistent results concerning the role of AQP4 in TBI. Taken together, variations in species, injury models, timing, methods, and targeted brain regions underscore the complexity of interpreting the role of AQP4 in TBI and account for some of the contradictory findings across studies.

##### AQP4 as a Therapeutic Target in Traumatic Brain Injury

A range of therapies targeting AQP4 have shown promise in reducing brain edema after TBI. Agents such as propofol, hypertonic saline, astaxanthin, and levetiracetam reduce AQP4 expression and associated inflammation or apoptosis [86,87,88,89]. *AQP4* siRNA consistently reduces edema and preserves AQP4 polarity [90,91,92]. Other agents, including acetazolamide, trifluoperazine, and ERK1/2 inhibitors, also prevent AQP4 mislocalization and edema progression [93,94,95]. Decompressive craniectomy and hypothermia normalize AQP4 levels and limit swelling [96,97]. Collectively, these findings highlight AQP4 as a promising therapeutic target in TBI. Table 4 lists the experimental studies that have focused on AQP4 as a therapeutic strategy in TBI models. None of the treatments presented in Table 4 act directly on AQP4 but rather modify the expression of AQP4, which target various upstream molecules.

#### 4.1.2. AQP4 in Ischemic Stroke

Experimental studies of AQP4 in ischemic stroke reveal its dynamic and region-specific role in edema development. Early after ischemia, AQP4 is upregulated at astrocyte end-feet, especially at the lesion border, where it promotes cytotoxic edema [102,103,104]. However, some studies have reported focal AQP4 loss in areas with high vascular permeability, suggesting complex temporal-spatial regulation [105,106]. *AQP4* knockout models have consistently shown reduced edema, infarct size, and BBB leakage and improved outcomes [65,66]. Hypertonic saline or genetic deletion of perivascular AQP4 has shown tomodulate water movement and therapy response [107,108]. Inflammatory stimuli such as IL-1α and comorbidities such as hyperglycemia alter AQP4 expression or polarity, worsening edema [109,110]. Overall, AQP4 is a critical, yet context-dependent, regulator of stroke-associated brain swelling.

Table 5 lists the experimental studies on AQP4 expression in ischemic stroke models.

##### Methodological Differences in Experimental Studies Regarding the Role of AQP4 in Ischemic Stroke

Experimental models of ischemic stroke vary widely in species (mice, rats), type of ischemia (transient vs. permanent middle cerebral artery–MCAO), duration of occlusion and reperfusion, and developmental stage of the animals. These methodological differences contribute to heterogeneity in AQP4-related findings. For example, early upregulation of AQP4 post-ischemia is frequently observed in transient models [102,104], whereas permanent MCAO often results in region-specific accumulation or loss of AQP4 [111,113]. The reperfusion phase plays a pivotal role: short durations (e.g., 30 min) result in AQP4 loss in vulnerable areas [105], whereas longer periods (23 h) reveal the protective effects of *AQP4* knockout on infarct volume and edema [65]. Neonatal versus adult animals further influence AQP4 dynamics due to immature BBB in neonates [103].

##### AQP4 as a Therapeutic Target in Ischemic Stroke

Therapeutically, diverse interventions target AQP4 to mitigate edema and improve outcomes. Statins [115], hyperosmolar therapies (e.g., TGN-020 and acetazolamide) [116,117], and antioxidants (edaravone) [118] reduce AQP4 expression and brain swelling. Approaches range from preconditioning (exercise, remote ischemia) [119] to post-conditioning (propofol, normobaric oxygen) [120,121]. Gene- and RNA-based strategies, including the use of siRNAs and microRNAs (e.g., miR-145 and miR-29b), downregulate AQP4 and reduce infarct size and apoptosis [122,123,124]. Together, these findings underscore AQP4 as a context-dependent but promising therapeutic target in ischemic stroke. The experimental studies focusing on AQP4 as a therapeutic strategy in ischemic stroke are listed in Table 6. Among the treatments referenced to in Table 6, only TGN-020 and AER-270/AER-271 are selective AQP4 inhibitors. All other molecules modify AQP4 expression, targeting various upstream molecules.

##### Critical Assessment of Studies Concerning AQP4 in TBI and Ischemic Stroke

- Shared Mechanisms of AQP4 in TBI and Ischemic Stroke in Experimental Studies

Across both TBI and AIS, AQP4 plays a dual role in edema regulation, participating in both cytotoxic and vasogenic edema formation. In both contexts, AQP4 is dynamically regulated and exhibits region- and time-dependent expression patterns. For instance, early upregulation of AQP4 in perivascular astrocyte end-feet is commonly observed in the acute phase after injury and is associated with increased water accumulation and cytotoxic edema [75,102]. AQP4 expression is also correlated with edema severity and is a potential biomarker of injury progression and outcome [65,81]. Moreover, in both TBI and stroke, the loss of AQP4 polarization, rather than total expression changes alone, appears to be critical in disrupting water clearance mechanisms and exacerbating edema [59,77].

- Divergent Mechanisms of AQP4 in TBI and Ischemic Stroke in Experimental Studies

Despite these similarities, divergent mechanisms arise on the of the nature of the insult. In TBI, some studies report global downregulation of AQP4 after injury [73,74], whereas others note upregulation driven by transcription factors such as Foxo3a [76], highlighting heterogeneity in responses potentially tied to injury severity and type (e.g., CCI vs. ballistic trauma). In contrast, ischemic stroke more consistently shows early AQP4 upregulation during cytotoxic edema and later regional loss during scar formation or prolonged ischemia [106,112]. Stroke models also demonstrate greater sensitivity to inflammatory modulation, such as the effects of IL-1α on AQP4 expression [109] and responses to systemic factors such as hyperglycemia [110]. Furthermore, genetic deletion of *AQP4* leads to beneficial effects in both models, reducing edema and lesion size, and improving outcomes [66,80], although stroke models uniquely show adaptive vascular remodeling with *AQP4* knockout [108]. Finally, the temporal dynamics differ: TBI models often exhibit early but transient changes in AQP4, whereas ischemic stroke involves biphasic or prolonged responses, depending on the reperfusion status [65,102].

#### 4.1.3. AQP4 in Experimental Subarachnoid Hemorrhage

The correlation between AQP4 and SAH has been demonstrated in numerous experimental studies, which revealed that AQP4 plays a significant role in early brain injury and late-onset cytotoxic edema.

Upregulated AQP4 is consistently observed in animal models of SAH, particularly in astrocyte end-feet around blood vessels and perivascular regions of the brain [143,144,145,146,147]. Its role in brain edema has also been established, as AQP4 contributes to both the formation and resolution of brain edema after SAH. In the early stages (acute phase), it facilitates the cytotoxic swelling of astrocytes, whereas in the later phases, it may support vasogenic edema resolution via interstitial fluid clearance [148]. Studies in *AQP4* knockout mice have shown more significant brain edema than in wild-type mice, followed by increased ICP and worsened neurological deficits [149]. In another study, compared with wild-type mice, *AQP4*-null mice presented decreased blood diffusion from the perivascular space to the brain parenchyma after SAH; however this phenomenon did not ameliorate the neurological deficits and neuroinflammation caused by SAH [150].

The glymphatic system (GS), a glia-dependent waste clearance pathway, is responsible for draining metabolic waste products and toxic factors from the brain. SAH has been shown to lead to redistribution or loss of AQP4 polarization, disrupting the glymphatic clearance pathway [151]. This leads to the accumulation of neurotoxic waste, contributing to delayed brain injury. *AQP4* knockout rodent models have been shown to exacerbate GS damage, brain edema, and neurological deficits after SAH [146,152]. Restoring AQP4 function or polarization may help reactivate glymphatic clearance and reduce secondary injury.

Table 7 lists the results of the studies on AQP4 expression in experimental SAH.

##### Methodological Differences Among Experimental Studies Regarding the Role of AQP4 in Subarachnoid Hemorrhage

Most studies report increased AQP4 expression in the early phase (within 24–72 h) after SAH, particularly in perivascular astrocyte end-feet [143,144,145,146,147]. This is thought to contribute to cytotoxic edema by promoting water influx into astrocytes and disrupting the glymphatic system, impairing waste clearance. In some models, later phases (days 3–7) may normalize or even reduce AQP4 expression, possibly due to astrocyte damage or death [148]. Experimental models of SAH also vary widely in species (mice, rats) and type of hemorrhage (perforation model vs. prechiasmatic cistern injection). In perforation models, AQP4 is markedly upregulated around blood vessels and in the cortex and hippocampus early post-SAH [143]. In prechiasmatic cistern injection models, AQP4 expression increases in the cortex and basal brain regions, and the polarization of AQP4 is disrupted, impairing glymphatic clearance [146,151]. The loss of AQP4 polarity is associated with impaired water clearance and worse outcomes. Some studies interpret increased AQP4 expression as detrimental, without assessing whether it is correctly localized. Mislocalized AQP4 may fail to perform its clearance function. AQP4 is highly expressed in astrocytes. *AQP4* knockout can disrupt astrocytic volume regulation, ionic homeostasis, and glutamate clearance, potentially worsening injury [149]. Hence, in *AQP4* knockout models, compensatory changes can introduce confounding effects.

##### AQP4 as a Therapeutic Target in Subarachnoid Hemorrhage

These findings highlight the potential of targeting AQP4 as a promising therapeutic agent. To this end, numerous studies have demonstrated that indirect downregulation of AQP4 by various approaches results in edema reduction [120,153,154,155,156,157,158,159,160]. One study demonstrated that glutamate elevated both AQP4 and edema [161]. Notably, none of these approaches include AQP4-specific inhibitors.

Table 8 includes the studies that have explored AQP4 as a therapeutic target in experimental SAH.

#### 4.1.4. AQP4 in Experimental Intracerebral Hemorrhage

AQP4 also plays a key role in ICH, as numerous studies over the past 20 years have revealed. AQP4 is upregulated in perihematomal regions within hours after ICH onset [163,164,165]. *AQP4* deletion exacerbated ICH-induced damage, leading to increased edema formation, BBB disruption, and increased neuronal death [166,167,168,169,170,171]. The results of these studies suggest that AQP4 plays a crucial role in the development of cytotoxic edema, while also contributing to the maintenance of BBB integrity and tight junction stability [172]. Furthermore, AQP4 is redistributed or depolarized, improving GS function [173].

Table 9 presents the findings of the studies mentioned above.

##### Methodological Differences Among Experimental Studies Regarding the Role of AQP4 in Intracerebral Hemorrhage

In ICH, the conflicting findings of some studies might be due to the dual role of AQP4 in brain edema; in the acute phase after ICH, upregulated AQP4 facilitates water influx into astrocytes, contributing to cytotoxic edema. In this context, AQP4 is detrimental [163]. In the subacute to chronic phases, AQP4 also facilitates the clearance of excess interstitial fluid and metabolic waste via the glymphatic system in a beneficial way [173]. Hence, whether AQP4 is protective or harmful depends largely on the time point studied. Inhibiting AQP4 too long or too late may impair recovery by disrupting fluid clearance. The differences in the experimental models used could also account for the observed differences. ICH models (e.g., collagenase vs. autologous blood injection) exhibit different degrees of injury severity and edema patterns. These variations influence AQP4 expression, localization, and the type of edema (cytotoxic vs. vasogenic), affecting the interpretation of the role of AQP4.

##### AQP4 as a Therapeutic Target in Intracerebral Hemorrhage

The findings of experimental ICH models clearly indicate that AQP4 may serve as a promising therapeutic agent. To this end, many studies have demonstrated that non-specific inhibition or downregulation of AQP4 using various molecules results in reduced edema in rodent ICH models [171,175,176,177,178,179,180,181,182,183]. Naturally occurring compounds have also been investigated in experimental ICH. Data show that their administration results in decreased AQP4 and brain edema [184,185,186,187,188,189,190,191,192]. Conflicting results have also been presented. Increased AQP4 levels have been linked to reduced brain edema after ICH [193,194,195,196,197], whereas AQP4 downregulation aggravated BBB permeability aggravation and worsened brain edema [198].

Cell transplantation has also been explored as a therapeutic target in experimental ICH. These interventions result in the downregulation of AQP4 and edema reduction [199,200,201,202].

After ICH, oxidative stress is aggravated, AQP4 is increased yet depolarized, and BBB permeability is lost. These effects were reversed with edaravone, an oxygen free radical scavenger, and MMP9-IN-1, an MMP9 inhibitor [203]. The results of the study suggested that mitigating the loss of AQP4 polarity alleviated brain edema and maintained BBB integrity [203].

Table 10 lists studies that have examined the therapeutic potential of AQP4 in experimental ICH.

### 4.2. AQP2

While AQP2 is prominently expressed in the kidneys, where it plays a critical role in regulating water homeostasis [206], its expression is also observed in other tissues. Studies have detected AQP2 expression within the CNS in the ependymal cell layer, subcortical white matter, and hippocampus. In contrast, in the peripheral nervous system, AQP2 seems to be involvedolved in pain and nerve damage responses, as indicated by its presence in structures such as the rat extra-temporal facial nerve, sensory neurons, and trigeminal ganglion neurons [207,208]. Furthermore, AQP2 expression has been detected in rat glioma cells, astrocytes, and microglial cell lines, whereas its expression was notably low in rat brain microvascular endothelial cells [21]. Unlike studies on AQP4, few experimental studies have explored the role of AQP2 in brain injury models.

#### 4.2.1. AQP2 in Traumatic Brain Injury

In addition to the expression of catalase and the receptor for advanced glycation end-products (RAGE), the expression of AQP2 has been shown to increase post-TBI. Metallothionein I and II *(Mt1+2)* knockout mice presented lower AQP2 expression post-injury but maintained increased levels of catalase and RAGE, thus indicating increased oxidative stress and inflammation [209].

#### 4.2.2. AQP2 in Intracerebral Hemorrhage

Microglial and astrocyte activation and induced cytokine secretion are major contributors to secondary brain injury following intracerebral hemorrhage. Post-injury, AQP2 expression is increased in the perihematomal area of the hemorrhaged rat brain, where it co-localizes with astrocytes and microglia. Moreover, targeted regulation of astrocyte AQP2 expression has led to divergent events. The overexpression of AQP2 promoted astrocyte activation and microglial migration, and both processes were inhibited by AQP2 silencing [21].

#### 4.2.3. AQP2 in Inflammation

During acute inflammation, AQP2 protein levels increase in the trigeminal ganglion of mice. The increased membrane expression of AQP2 was universal, whereas its cytoplasmic expression increased only in small neurons. This redistribution could raise the possibility of an adaptation to nociceptive conditions or a consequence of inflammation [210].

Table 11 lists the experimental studies on AQP2 in experimental brain injury models.

The current body of research investigating the role of AQP2 in acute BI models is limited. Although several investigators have obtained intriguing data regarding AQP2 expression and function post-brain injury, key aspects of its role in pathomechanisms are lacking, thus restricting our ability to fully delineate its implications. Further studies are needed to clarify the involvement of AQP2 in brain injury.

### 4.3. AQP9

Another member of the aquaporin family is AQP9, an aquaglyceroporin expressed throughout the brain that has extensive functions. Its dual role in water and glycerol transportation may be associated with both brain water movement and the regulation of neuronal metabolism.

#### 4.3.1. AQP9 in Traumatic Brain Injury

AQP9 protein and mRNA upregulation has been observed in both the ipsilateral parietal cortex and the hippocampus. Increases in aquaporin expression correlate with alterations in brain water content [213,214]. Selective targeting of AQP9 and its regulator HIF-1α reduced brain edema and glycerol levels in the extracellular space [211,212,215]. In contrast, increased injury severity has been associated with reduced AQP9 mRNA and protein expression levels [74]. Ethanol and agmatine (a guanidine compound) downregulated AQP9 expression levels post-injury [216,217].

#### 4.3.2. AQP9 in Ischemic Stroke

Significant swelling of the ischemic hemisphere accompanies remarkable increases in AQP9 protein expression [102,218]. Increased AQP9 levels have been reported to be associated with elevated expression of matrix metalloproteinase (MMP) family members [220]. Inhibition of either HIF-1α or p38 mitogen-activated protein kinase (MAPK) resulted in decreased AQP9 expression levels [219,221]. Ethanol administration and pre-conditioning with flurbiprofen axetil downregulated AQP9 expression [220,222].

#### 4.3.3. AQP9 in Intracerebral Hemorrhage

Compared with wild-type mice, *AQP9*-null mice presented impaired neovascularization and greater neurological deterioration [223]. However, another study demonstrated that increased AQP9 expression in the hippocampus of diabetic mice post-ICH was negatively correlated with brain angiogenesis, neuronal survival, and BBB function [224]. Naturally occurring compounds, including recombinant hirudin and curcumin, have been investigated in experimental ICH. Their administration led to a reduction in AQP9 [177,185].

#### 4.3.4. AQP9 in Hyperosmotic Stress

Cultured rat astrocytes presented increased AQP9 mRNA and protein levels after mannitol-induced hyperosmotic stress, but this increase did not involve de novo protein synthesis. Under hyperosmotic conditions, suppression of p38 MAPK downregulated AQP9 expression, pinpointing a regulatory connection that paves the way for therapeutic approaches [225].

Table 11 lists the experimental studies on AQP9 in experimental brain injury models.

##### Methodological Differences Among Experimental Studies Regarding the Role of AQP9 in Acute Brain Injuries

AQP9 plays a significant role in brain function as the transportation of water, glycerol, and lactate not only regulates water homeostasis but also promotes metabolic processes in the brain. It is evident that post-brain injury, the expression of AQP9 varies and depends on the brain location studied and the experimental model used [74,211]. In addition, AQP9 expression is affected post-injury by numerous signaling pathways, including the HIF-1α, MAPK, and NF-κB pathways, thus highlighting the complexity governing the regulation of these molecules [74,211,212,215,219,221].

### 4.4. AQP11

Aquaporin-11 (AQP11) is a member of the aquaporin family of water channel proteins that facilitate water transport across cell membranes. While other aquaporins, such as AQP4, have been extensively studied in the context of brain function and injury, the expression and specific role of AQP11 in the brain remain poorly understood.

A transcriptomic analysis revealed *AQP11* in the human cortex and hippocampus, alongside *AQP1*, *AQP4*, and *AQP9*, which are known to be expressed in the mammalian brain. Nevertheless, the localization of the AQP11 protein remains unknown [227]. A limited number of experimental studies investigating the role of AQP11 in brain injury models have been reported.

#### 4.4.1. AQP11 in Ischemic Stroke

The primary role of AQP11 in the lens and kidney is to transport hydrogen peroxide (H_2_O_2_), thereby exerting a protective effect. Oxidative stress resulting from accumulated H_2_O_2_ is recognized as an underestimated neuropathogenic factor [228].

Neuroinflammation and hypoxia are characteristics of stroke [229,230], among other conditions. These conditions can increase oxidative stress and exacerbate pathological outcomes [231,232]. Established cell lines for astroglia and neurons were utilized to monitor changes in the transcript levels of human AQPs (*AQP0* to *AQP12*) in response to inflammation and hypoxia, which revealed upregulated *AQP11* transcripts in both cell lines [35]. Furthermore, enhancing peroxiporin expression through LPS pretreatment reduced subsequent H_2_O_2_-induced malondialdehyde (MDA) responses compared with those in controls [35]. MDA assays are employed to quantify lipid peroxidation levels following brief exposure to H_2_O_2_.

The protective role of AQP11 against elevated H_2_O_2_ levels is attributed to its high expression in the endoplasmic reticulum, with an additional presence in the plasma membrane. It has been proposed that AQP11 facilitates the export of H_2_O_2_ from intracellular organelles into the cytoplasm, followed by its removal into extracellular fluid compartments, thereby mitigating the oxidative stress associated with metabolic activity. Researchers suggest that AQP11 may play a similar protective role in neural and glial cells, highlighting the potential clinical significance of peroxiporins across multiple organ systems [35].

#### 4.4.2. AQP11 in Intracerebral Hemorrhage

In a rat model of collagenase-induced ICH, administration of the miR-27a-3p mimic reduced brain edema, BBB disruption, and neuronal loss by suppressing AQP11 upregulation in perihematomal tissue and brain endothelial cells. These findings suggest that miR-27a-3p protects the BBB and mitigates brain injury by targeting endothelial AQP11. Given its downregulation in ICH patients serum, miR-27a-3p may hold therapeutic potential, although its exact role in clinical ICH remains to be fully defined [226].

Table 11 lists the studies on AQP11 in experimental brain injury models.

While AQP11 is expressed in the brain and may influence water transport mechanisms, its specific role in brain injury has not been revealed. Further research is needed to elucidate its potential involvement in neuropathological conditions and possible therapeutic implications.

## 5. Aquaporins as Targets in Brain Injuries—Translational Perspectives

### 5.1. Roles of AQPs in Clinical vs. Experimental Acute Brain Injuries

#### 5.1.1. Traumatic Brain Injury

Clinical studies generally point toward consistent upregulation of AQP4 following TBI, which is often linked to edema development and neuroinflammation. In contrast, experimental studies have shown heterogeneous or even contradictory patterns of AQP4 expression depending on the model, species, and time point. In some models, AQP4 expression decreases globally [73,74], whereas in other models AQP4 expression is upregulated [75,76]. Furthermore, some studies emphasized disrupted AQP4 polarity, rather than absolute expression, as a driver of edema severity [77,78]. Another contrast lies in regional and temporal specificity: clinical studies typically detect systemic or persistent AQP4 increases (e.g., in CSF, plasma), whereas experimental data often reveal biphasic patterns, regional variation, or opposite trends in core vs. peripheral brain zones [84,85]. While clinical studies largely support the pathological role of AQP4 overexpression in TBI, experimental data reveal a more complex and dynamic picture, with model-specific variations in AQP4 expression, localization, and regulation. Experimental studies have highlighted the dual role of AQP4 in TBI; AQP4 contributes to early cytotoxic edema by allowing excessive water influx into astrocytes due to ionic imbalances and BBB disruption. This leads to the swelling of brain tissue, increased ICP, and further neuronal injury. In later stages, AQP4 facilitates vasogenic edema clearance by enhancing glymphatic drainage and CSF circulation, which helps to reduce swelling and improve recovery [17]. Hence, understanding the precise timing and regulation of AQP4 expression in TBI is critical for developing therapeutic strategies to modulate AQP4 activity to minimize harmful effects while promoting beneficial fluid clearance.

AQP2 is known primarily for its function in the kidneys, but it is also expressed in brain regions related to vasopressin signaling and CSF dynamics [20,22]. In clinical and experimental studies, AQP2 has been shown to increase consistently, and it is thought to exacerbate fluid retention and increase ICP [22]. AQP9 helps neurons and glial cells adapt to metabolic stress by providing alternative substrates for energy production [19,20]. Although not studied in the clinical setting, the results of the experimental studies have shown that AQP9 inhibition helps ameliorate brain edema and neuronal damage, and improves neurobehavioral outcomes post-TBI [212]. In experimental studies, AQP11 has been shown to play a potential role protecting against oxidative stress-induced secondary injury [20].

#### 5.1.2. Ischemic Stroke

Clinical studies suggest that AQP4 plays a critical role in edema formation and resolution in acute ischemic stroke, with a focus on its diagnostic and prognostic utility. Clinically, serum AQP4 levels have emerged as potential biomarkers for infarct size and recovery. Genetic associations have also been identified [62]. In contrast, experimental studies offer a more detailed and sometimes conflicting picture. Many animal models have confirmed that AQP4 is upregulated during the early stages of ischemia. However, some models have reported regional or temporal AQP4 loss, especially in areas with increased vascular permeability [105] or in the striatal core at 24 h post-MCAO [106], suggesting that AQP4 disruption may worsen or delay edema resolution. Experimental models also enabled functional testing through genetic manipulation. *AQP4* knockout mice presented smaller infarcts, less edema, and improved recovery [65,66]. Chronic *AQP4* deletion altered BBB water exchange and increased capillary density [108], whereas the absence of perivascular AQP4 impaired the efficacy of hypertonic saline [107]. In summary, while clinical studies link AQP4 to prognosis, edema severity, and genetic susceptibility, experimental models provide a deeper understanding of its biphasic role, regional variability, and therapeutic potential, highlighting both protective and pathological effects in ischemic stroke. More specifically, AQP4 upregulation during the acute phase exacerbates edema formation, worsening neurological outcomes. In contrast, at later stages, AQP4 assists in clearing excess fluid and promoting brain recovery [233].

Only one clinical study on AQP2 in ischemic stroke has been conducted [64]. These results indicate that during ischemic stroke, vasopressin levels increase, potentially leading to the upregulation of AQP2, which could contribute to cerebral edema by promoting excessive water retention in the brain [234]. Although AQP2 is not a primary aquaporin involved in ischemic stroke, it may contribute to vasopressin-mediated cerebral edema and CSF disturbances following ischemic injury [10]. AQP9 supports energy metabolism in neurons and astrocytes affected by ischemia and vasospasm. It contributes to neuroinflammation and oxidative stress, which can influence secondary injury progression [27]. Increased AQP9 expression facilitates energy production by providing alternative metabolic substrates in ischemic areas, facilitating neuron survival [235]. In clinical studies, genetic associations have been identified that are associated with increased edema [62] or with stroke risk in hypertensive patients [63]. In experimental studies, upregulated AQP9 seems to exacerbate injury by enhancing astrocyte swelling and supporting neurotoxic metabolite flux [218,219,220]. To the best of our knowledge, there are no clinical studies of AQP11 in the context of ischemic stroke. Experimental studies suggest that AQP11 may help mitigate oxidative stress and neuronal apoptosis [35].

#### 5.1.3. Subarachnoid Hemorrhage

In the clinical context, upregulated expression of AQP4 is noted, with polarization loss at astrocytic end-feet possibly impairing glymphatic clearance and contributing to prolonged edema and neuroinflammation [42]. The findings from ample experimental studies on subarachnoid hemorrhage indicate that in the acute phase of SAH, increased AQP4 expression contributes to cerebral edema, inflammation, and neuronal apoptosis, while it plays a role in exacerbating or mitigating edema depending on the stage of injury [148]. Experimental models have shown that AQP4 inhibition is most effective early (within hours) after SAH to reduce cytotoxic edema [81,153,154]. This highlights a potential therapeutic window in patients when AQP4-targeted therapies might be beneficial. In patients with SAH, brain edema and elevated ICP are major contributors to early brain injury. Since AQP4 plays a critical role in water transport, modulating its function could help reduce edema and optimize ICP management, especially when traditional methods are insufficient. SAH is associated with impaired glymphatic clearance due to blood breakdown products and astrocyte dysfunction [146]. Animal studies showing AQP4 depolarization after SAH suggest that this may also occur in the clinical setting. Therefore, therapies aimed at restoring AQP4 polarity or function may be as important as modulating its overall expression. To the best of our knowledge, no other aquaporins have been studied in the context of SAH.

#### 5.1.4. Intracerebral Hemorrhage

Human genetic studies have shown that *AQP4* variants are associated with increased hematoma and perihematomal edema volume [70] and that *AQP4* SNPs may influence ICH susceptibility [71]. Experimental studies have shown that AQP4 contributes to PHE in the context of ICH by regulating water influx into surrounding tissues. During the inflammatory response, AQP4 influences neuroinflammatory cascades that contribute to secondary injury. Experimental models have shown that AQP4 inhibition is beneficial early (within 6–12 h) post-ICH but potentially harmful if prolonged (due to impaired fluid clearance) [165]. AQP4-modulating drugs might serve as adjunct therapies to reduce ICP and tissue swelling in the early phase of ICH. The dual role of AQP4 in edema formation and clearance suggests that targeted AQP4 modulation could be a therapeutic strategy for ICH management [169,236]. Any clinical AQP4-targeted therapy needs to be time-restricted and possibly administered within a defined therapeutic window.

Both clinically and experimentally, AQP2 has emerged as a potential systemic biomarker with prognostic value in ICH. AQP2 upregulation has been reported to be associated with cytotoxic edema and astrocytic swelling, potentially regulated by vasopressin or other injury-induced pathways [21]. While data on AQP9 are limited, its role in metabolic adaptation may also be relevant in the hemorrhagic context, as shown by experimental studies. AQP9 is negatively correlated with brain angiogenesis, neuronal survival, and BBB function [224] and may contribute to metabolic adaptation and neuroinflammation [236]. AQP11 could play a role in reducing oxidative stress, supporting cellular repair, and resolving inflammation, although further research is needed [237,238].

Collectively, these observations suggest a complex spatial and temporal pattern of aquaporin dysregulation shaped by both central and systemic injury dynamics. The clinical findings outlined above reveal both the promise and complexity of targeting aquaporins in acute brain injury. Experimental studies provide critical mechanistic insights into these roles and help clarify the temporal, regional, and molecular influences that shape aquaporin function under pathological conditions.

### 5.2. Mechanisms of AQP Regulation in Acute Brain Injuries

AQPs contribute to both cytotoxic and vasogenic edema. AQP4 upregulation and loss of polarity after injury impair glymphatic clearance and exacerbate swelling [239]. The regulation of AQP expression is multifactorial. Inflammatory mediators such as TNF-α and IL-1β modulate AQP expression and redistribution, particularly increasing AQP4 and AQP9 in reactive astrocytes [86]. Hypoxia and ischemia activate transcriptional regulators such as HIF-1α, which can upregulate AQP4 and AQP9, facilitating both water and solute movement in damaged tissue [211]. Oxidative stress alters protein trafficking and AQP stability, disrupting their membrane localization and function [237]. MicroRNAs provide post-transcriptional regulation: miR-320 and miR-130a target AQP4 [240], and miR-27a-3p suppresses AQP11 expression in endothelial cells, maintaining BBB integrity after ICH [226]. Anchoring proteins, such as α-syntrophin, are critical for maintaining AQP4 polarity; their loss leads to AQP mislocalization and dysfunctional glymphatic flow [107].

These regulatory pathways highlight the therapeutic potential of precisely targeting AQP expression and localization to reduce edema, preserve BBB integrity, and improve neurological outcomes.

### 5.3. Targeting Aquaporins in Acute Brain Injuries: Perspectives and Limitations

#### 5.3.1. Therapeutic Strategies

Therapeutic strategies aimed at modulating AQP function include specific and non-specific inhibitors, gene silencing, and RNA-based therapies. Naturally occurring inhibitors, such as TGN-020, selectively inhibit AQP4 and have shown efficacy in preclinical models by reducing brain edema. However, their off-target effects and insufficient BBB penetration limit their translational potential. Gene silencing techniques, including siRNAs and antisense oligonucleotides that target AQP expression directly, constitute another alternative therapeutic approach. However, these approaches face challenges with respect to carrier systems, immunogenicity, and transient vs. permanent effects.

#### 5.3.2. Therapeutic Limitations

Targeting AQPs in the context of brain injuries has emerged as a promising therapeutic strategy, particularly in managing edema formation and resolution, CSF dynamics, and BBB dysfunction. However, the approach is complex due to the delicate balance of water transport, inflammation, and neuroprotection. The results of these experimental studies suggest that AQP4 plays a dual and dynamic role in the pathophysiology of brain injury. Its upregulation is associated with edema, inflammation, and impaired waste clearance. Therapeutic targeting of AQP4 (especially in early stages) shows promise in experimental brain injury models but requires precise timing and further validation. Restoration of AQP4 polarity or enhancement of glymphatic flow may be as important as modulating its overall expression. However, clinical translation will require selective drugs, precise timing, and biomarkers to monitor AQP4 activity in patients.

AQP4 may help control cerebral edema. In the context of cytotoxic edema (e.g., ischemic stroke), AQP4 facilitates water entry into swollen astrocytes. Inhibition may reduce edema. In vasogenic edema (e.g., trauma), AQP4 may help clear excess interstitial fluid, suggesting that activation might be beneficial in these cases. This duality offers a therapeutic window depending on the type and phase of brain injury. The limitations and challenges lie in the dual role of AQP4. AQP4 can either exacerbate or relieve edema, depending on the context (cytotoxic vs. vasogenic). The timing of intervention is also critical; early inhibition vs. late activation requires precise diagnostic tools and delivery timing. In ICH, AQP4 expression and polarity changes could serve as biomarkers for edema severity, glymphatic dysfunction, and prognosis or treatment response.

Another major issue is the lack of selective and safe inhibitors. Most AQP modulators lack selectivity or BBB penetration or have toxic effects. For example, TGN-020 is a promising AQP4 inhibitor in animals but is not approved for clinical use because of its off-target effects and delivery barriers. Mercury-based inhibitors are toxic and non-selective. Delivering AQP-targeted therapies across the BBB remains technically difficult, especially in patients where the BBB is only mildly disrupted. Inhibition of AQP4 may lead to compensation by other channels (e.g., AQP1 and ion channels), potentially diminishing therapeutic effects or causing unintended shifts in water homeostasis. Finally, AQP4 is important for normal brain water homeostasis, potassium buffering, and waste clearance. Chronic inhibition may cause long-term harm, especially in non-acute conditions.

Data from experimental animal models support the development of BBB-penetrant AQP4-targeting drugs for clinical testing. The current limitations are that AQP4 is not yet clinically targetable due to the lack of safety, the complexity of the dual role of AQP4, the poor understanding of the human glymphatic system, delivery challenges, and the risk of off-target effects. There is a need for real-time monitoring tools for edema and AQP4 status. Clinical trials could be designed to test the safety and efficacy of early-phase AQP4 inhibition. Finally, patient stratification may help identify responders.

AQP2 is a peripheral water channel with limited but emerging relevance in brain injury. Although experimental data are sparse, interest in AQP2 as a therapeutic or diagnostic target in brain injury is growing. Serum and CSF vasopressin levels, which regulate AQP2 in the kidney, are often altered in ABI, suggesting indirect relevance. However, more direct mechanistic studies are needed to establish its function in the brain and to assess its feasibility as a clinical target. AQP2 is not a direct CNS target, and its effects are systemic and not localized to brain injury zones; thus, targeting AQP2 may have limited utility beyond supportive management of water/electrolyte imbalance.

Although still in the early stages of translational research, AQP9 shows promise as a dual metabolic and inflammatory target in brain injury. AQP9 could also potentially serve as a biomarker of injury severity or energy imbalance. However, there is a lack of selective AQP9 inhibitors, a limited understanding of the dual role of AQP9 (protective vs. harmful), and no clinical trials exploring AQP9 targeting. Targeted modulation could refine therapeutic approaches in acute brain injuries.

AQP11 could also be used as a novel intracellular target in brain injury, particularly in addressing ER stress-induced cell death and neuroinflammation. Emerging evidence suggests that it has a potential neuroprotective role in ER stress responses. AQP11 is upregulated in endothelial cells post-ICH and may contribute to BBB breakdown. Hence, targeting AQP11 in the cerebral endothelium may offer a new strategy to preserve BBB integrity in acute brain injuries. However, our understanding of the functions of AQP11 is limited. It remains unclear how AQP11 contributes to BBB dynamics in brain injury patients since AQP11 expression in human brain tissues post-injury is poorly characterized. The lack of selective AQP11 inhibitors means that none of the existing drugs can specifically target AQP11 safely. Finally, AQP11 is an intracellular aquaporin, increasing the complexity of direct drug targeting.

The most promising and studied target is AQP4 because of its central role in edema and fluid dynamics. AQP2 has indirect clinical relevance, whereas AQP9 and AQP11 have experimental potential for metabolic and inflammatory modulation. While experimental studies have yielded encouraging results in modulating AQPs in brain injury models, key challenges remain. Table 12 summarizes the perspectives and challenges of targeting AQPs as therapeutic targets.

#### 5.3.3. Future Directions

Despite promising preclinical data, no AQP-targeting therapies have reached clinical use in the treatment of brain injuries. The development of isoform-specific modulators, advanced imaging tools to assess AQP function, and clinical studies are essential. Integrating AQP-targeted approaches with imaging and biomarker-guided strategies may enhance therapeutic precision and outcomes.

## 6. Conclusions

Aquaporins, particularly AQP4, are integral to maintaining water homeostasis in the brain but also contribute to the pathophysiology of traumatic and non-traumatic brain injuries. Their roles in edema formation, glymphatic clearance, metabolic adaptation, oxidative stress, and neuroinflammation make them potential therapeutic targets. Further research into the precise regulation of aquaporins could lead to novel treatment strategies for conditions such as TBI, ischemic stroke, aSAH, and ICH, ultimately improving neurological outcomes. Experimental studies provide strong rationales for clinical translation, although challenges remain. Major challenges include drug specificity, timing of intervention, and delivery across the BBB. Integrating experimental results with clinical strategies may lead to targeted therapies that reduce edema and improve neurological recovery.

## Figures and Tables

**Table 1:** Localization and function of AQP2, 4, 9, and 11 in the human brain.

Characteristic	AQP2	AQP4	AQP9	AQP11
Location	Kidney collecting ducts, minimal expression in the brain astrocytes [22]	Astrocyte end-feet, ependymal cells [11,12,13,14]	Astrocytes, neurons [18]	Endoplasmic reticulum (ER) of astrocytes and glial cells [24]
Primary Function	Water reabsorption in response to ADH [21]	Water transport, brain water homeostasis [11,12,13,14,15]	Glycerol, lactate, and water transport [17]	Intracellular water regulation, ER stress response [24]
Role in brain injury	Indirect role via systemic water balance, affected by SIADH or DI [23]	Regulates brain edema formation and resolution [25]	Supports metabolic adaptation by shuttling lactate and glycerol for energy [19,20]	Protects against ER stress and oxidative damage [24]
Involvement in edema	ADH-induced upregulation may exacerbate fluid retention and increase ICP [10,22]	Major contributor to cytotoxic and vasogenic edema [26]	Indirect role, may contribute to cell swelling [27]	Minimal role in extracellular edema formation
Inflammatory response	Limited direct involvement	Can exacerbate inflammation during edema [11,28]	May amplify inflammation through metabolic byproducts [29]	May reduce inflammation by alleviating ER stress [30,31,32]
Neuroprotection	Not directly involved in neuroprotection	Can be detrimental if overexpressed during injury [33]	Provides metabolic support for neurons [34]	May protect cells by maintaining ER function [35]
Therapeutic potential	Managed through ADH modulation in systemic disorders [23]	Target for edema management using AQP4 inhibitors [36]	Potential target for reducing inflammation and metabolic imbalance [37,38]	Investigational target for reducing ER stress-related damage [32,35]
Clinical relevance	Important in brain injury with secondary endocrine issues (e.g., SIADH, DI) [39]	Widely studied in stroke, TBI, and hemorrhages [40,41,42]	Relevant in ischemia and stroke [43]	Limited research, emerging interest in neuroprotection [44]

ADH, anti-diuretic hormone; AQP, aquaporin; DI, diabetes insipidus; ER, endoplasmic reticulum; ICP, intracranial pressure; SIADH, syndrome of inappropriate antidiuretic hormone secretion.

**Table 2:** Clinical studies investigating AQP2, 4, 9, and 11 in ABIs.

Disease	Aquaporin	Main Findings	Ref.
TBI	AQP4	AQP4 increases from 15 h to 8-days post-injury; strongly expressed in astrocytomas and peritumoral tissue.	[68]
	AQP4	AQP4 and VEGF co-expressed in astrocytes in edematous tissue; may regulate water flow.	[47]
	AQP4	No exon 4 mutation found in *AQP4* gene among TBI patients; further genetic studies needed.	[54]
	AQP4	CSF AQP4 elevated post-TBI; levels reflect water metabolism and correlate with ICP control.	[40]
	AQP4	HMGB1-TLR4 signaling in microglia promotes IL-6 release, increasing astrocytic AQP4 expression and edema.	[49]
	AQP4	SNPs in the *AQP4* gene (rs3763043) associated with 6-month outcome; influence recovery post-TBI.	[53]
	AQP4	Elevated AQP4 microparticles in TBI patient blood; potential non-invasive biomarker.	[55]
	AQP4	AQP4 upregulation peaks at 7–30-days post-TBI; associated with hypoxia and neuroinflammation.	[46]
	AQP4	NDEs show 8.9-fold AQP4 increase in acute mTBI; 3.6-fold in chronic mTBI; phase-specific biomarker.	[45]
	AQP4	Chronic TBI with CI shows elevated IL-6 and tau biomarkers; AQP4 not elevated in CI cases.	[50]
	AQP4	Increased AQP4 expression correlates with DAI severity and brain edema in CT and pathology.	[48]
	AQP4	miR-211-5p suppresses MMP9 and AQP4; reduced levels linked to increased AQP4 in TBI.	[52]
	AQP2, AQP4, AQP9	AQP2 levels correlated with chronic SDH volume and midline shift; no correlation found in acute cases.	[22]
	AQP4	Blast-related mTBI alters AQP4 expression and glymphatic function; associated with neurodegeneration.	[51]
AIS	AQP2	Elevated urinary AQP2 in hyponatremia post-stroke; not solely AVP-dependent.	[64]
	AQP4	AQP4 upregulation near ischemic foci linked to edema development via astrocytic transport routes.	[41]
	AQP4	The *AQP4* SNP rs9951307 is associated with reduced risk of severe brain edema.	[62]
	AQP4	White matter shows increased AQP4 expression and edema compared to cortex post-stroke.	[59]
	AQP4	Higher baseline serum AQP4 predicts reduced infarct growth and better recovery.	[60]
	AQP4	High SBP post-thrombolysis linked to AQP4 upregulation and early neurological deterioration.	[61]
	AQP9	*AQP9* SNPs affect stroke risk in hypertensive patients.	[63]
aSAH	AQP4	Upregulation of AQP4 in astrocytic processes; loss of polarization; no neuronal expression; implicated in edema dynamics.	[42]
ICH	AQP2	Lower serum AQP2 levels associated with worse 90-day outcomes; AQP2 overexpression promotes astrocyte activation and pro-inflammatory signaling.	[21]
	AQP4	AQP4 and thrombin contribute to cerebral edema; findings differ between humans and rats; need for human-based data.	[69]
	AQP4	The *AQP4* SNP rs1058427 is associated with increased hematoma and perihematomal edema volume.	[70]
	AQP4	*AQP4* SNPs may influence ICH susceptibility and age of onset, though findings did not remain significant after correction.	[71]

ABI, acute brain injury; AIS, acute ischemic stroke; AQP, aquaporin; aSAH, aneurysmal subarachnoid hemorrhage; AVP, arginine vasopressin; CI, cognitive impairment; CSF, cerebrospinal fluid; DAI, diffuse axonal injury; HMGB1, high mobility group box 1 protein; ICH, intracerebral hemorrhage; ICP, intracranial pressure; IL, interleukin; miR, microRNA; MMP9, matrix metalloproteinase-9; mTBI, mild traumatic brain injury; NDEs, neuron-derived exosomes; SBP, systolic blood pressure; SDH, subdural hematoma; SNP, single nucleotide polymorphisms; TBI, traumatic brain injury; TLR4, toll-like receptor 4; VEGF, vascular endothelial growth factor.

**Table 3:** Experimental studies investigating AQP4 in experimental TBI models.

Experimental Model	Main Findings	Ref.
Rat model of penetrating ballistic-like brain injury via rapid balloon inflation/deflation	Global *AQP4* mRNA decreased at 24 h; significant reductions in AQP4 M1 and isoform 3 at 3–7 days.	[74]
Rat CCI	Brain edema peaked at 24 h; global AQP4 protein expression was reduced by 48 h, despite only transient reductions in cortical perfusion.	[73]
Murine CCI	TBI triggered nuclear translocation of Foxo3a in astrocytes, which increased AQP4 expression, leading to cytotoxic edema and memory deficits; depletion of Foxo3a prevented AQP4 upregulation and rescued edema.	[76]
Adult male Wistar rat TBI model assessing hippocampal proteins	Hippocampal AQP4 increased starting at 1 h, peaking at 12 and 72 h, closely correlating with brain water content and edema severity.	[75]
Rat CCI-induced TBI with intraventricular siRNA infusion	In both mild and severe TBI, AQP4 expression increased in contralateral brain tissue over different time courses; *AQP4* knockdown reduced brain water content.	[81]
Murine CCI comparing *AQP4* knockout and wild-type mice	AQP4 deficiency reduced brain edema, intracranial pressure, and neuroinflammation; it improved BBB integrity, enhanced amyloid β clearance, and led to better cognitive outcomes.	[80]
In vitro FPI in cultured astrocytes	FPI induced a significant upregulation of AQP4 in the astrocyte plasma membrane via new protein synthesis; *AQP4* knockdown markedly reduced trauma induced astrocyte swelling.	[83]
Murine CCI comparing *AQP4*^+/+^ and *AQP4*^–/–^ mice	*AQP4*^–/–^ mice showed reduced injury volume, intracranial pressure, and brain water accumulation, as well as ultrastructural changes that contributed to improved neurological outcomes.	[79]
Rat TBI model of contusional injury	Early after TBI, AQP4 and DG maintained perivascular polarization; later, loss of polarization (with upregulation of AQP4 isoforms M1 and M23) correlated with severe cytotoxic edema.	[78]
Murine closed skull “Hit and Run” TBI model	Global AQP4 increased post-TBI, but a prominent loss of polarized localization at astrocyte end-feet peaked at 7-days, suggesting a compensatory mechanism for edema resolution.	[77]
Murine TBI model focusing on the hippocampal CA1 region with adenosine A2A receptor inactivation	TBI impaired perivascular AQP4 polarization in the hippocampal CA1 area; A2AR knockout alleviated these abnormalities, suggesting A2AR involvement in AQP4 dysregulation.	[56]
Ovine impact acceleration head injury model of closed head contusional injury	Within contusions, AQP4 expression was heterogeneous: some astrocytes in the core were non-viable (AQP4 negative), whereas pericontusional astrocytes showed robust AQP4 expression, suggesting regional differences in edema regulation.	[84]
Rat CCI-induced TBI with analysis of both injured and contralateral hemispheres	In the injured hemisphere, vasogenic edema occurred first followed by cellular edema (with AQP4 downregulated during vasogenic and upregulated during cellular edema); the contralateral side showed a delayed pathological progression.	[85]
Rat cortical contusion injury model with secondary insults (hypoxia and hypotension)	Secondary insults at 5 h post-injury significantly worsened BBB function and blunted the normal upregulation of AQP4, thereby exacerbating brain edema and ionic imbalance.	[82]

A2AR, adenosine receptor; AQP, aquaporin; BBB, blood–brain barrier; CCI, controlled cortical impact; DG, dystroglycan; FPI, fluid percussion injury; TBI, traumatic brain injury.

**Table 4:** AQP4 as a therapeutic target in experimental TBI.

Molecule/Intervention	Experimental Model	Main Findings	Ref.
Propofol	Rat CCI	Reduced brain edema, reduced AQP4 expression; blocked IL-1β/TNF-α-induced AQP4 via NF-κB/p38.	[86]
*AQP4*-siRNA	CCI in post-natal day-17 rats	Reduced edema, increased motor/cognitive recovery, reduced neuronal death, 30% reduction in AQP4 expression.	[90]
*AQP4*-siRNA	Rat TBI (unspecified method) + multimodal MRI	Reduced AQP4 expression and edema at 6–12 h post-TBI; validated MRI for edema tracking.	[91]
*AQP4*-siRNA	Rat TBI (unspecified method)	Prevented AQP4 polarity reversal (astrocytic vs. perivascular); reduced edema.	[92]
Magnesium sulfate	Rat diffuse TBI (impact-acceleration)	Restored AQP4 polarity (perivascular localization), reduced edema.	[98]
Acetazolamide	Murine/human astrocyte TBI models (unspecified)	Prevented AQP4 redistribution post-TBI; reduced cytotoxic edema.	[93]
Progesterone	Rat bilateral medial frontal cortex contusion	Reduced brain water content; region-specific AQP4 reduction (peri-contusion) and increase (third ventricle).	[99]
Levetiracetam	Rat FPI	Dose-dependent decrease in AQP4 mRNA/protein and edema; high dose most effective.	[89]
Phorbol dibutyrate	Rat diffuse TBI (unspecified)	Reduced brain water content and AQP4 upregulation post-TBI.	[100]
Intranasal delivery of nerve growth factor (NGF)	Rat TBI (weight-drop model)	Reduced brain edema, reduced expression of AQP4, IL-1β/TNF-α, and reduced apoptosis.	[101]
U0126 (ERK1/2 inhibitor)	Rat astrocyte scratch-injury model	ERK1/2 activation reduced AQP4; U0126 restored AQP4 levels.	[95]
Decompressive craniectomy (DC) + hypothermia	Murine TBI (unspecified) + MRI	DC + hypothermia reduced AQP4 expression and edema volume; AQP4 correlated with edema.	[97]
Decompressive craniectomy	Rat FPI	DC reduced cortical AQP4 expression and water content at 48 h post-TBI.	[96]
Trifluoperazine	Rat TBI (unspecified method)	Reduced AQP4 accumulation on astrocyte end-feet, reduced apoptosis/inflammation, increased recovery.	[94]
3% Hypertonic saline	Rat CCI	Reduced edema, AQP4, TNF-α, IL-1β, and caspase-3-mediated apoptosis.	[87]
Astaxanthin + Bumetanide	Murine CCI	Reduced edema, BBB disruption, and AQP4/NKCC1 expression; bumetanide reduced AQP4.	[88]

AQP, aquaporin; CCI, controlled cortical impact; DC, decompressive craniectomy; ERK, extracellular signal-regulated kinase; FPI, fluid percussion injury; IL, interleukin; MRI, magnetic resonance imaging; NF-κB, nuclear factor-kappa B; NGF, neural growth factor; NKCC1, Na-K-Cl cotransporter 1; siRNA, small interfering RNA; TBI, traumatic brain injury; TNF-α, tumor necrosis factor alpha.

**Table 5:** Experimental studies investigating AQP4 in ischemic stroke.

Experimental Model	Main Findings	Ref.
Murine model of transient focal cerebral ischemia (occlusion followed by reperfusion)	Two peaks of maximal hemispheric swelling were observed at 1 h and 48 h after ischemia. At 1 h, AQP4 expression was significantly increased on astrocyte end-feet in both the core and border of the lesion; at 48 h, AQP4 was elevated throughout astrocytes in the border.	[102]
Rat model of neonatal stroke (using high-field 11.7 T MRI and immunohistochemistry)	At 24 h, MRI findings indicated edema, coinciding with significant increases in AQP4 expression on astrocytic end feet in the lesion border. At 72 h, imaging findings persisted with a slow normalization of AQP4 in the border, and by 28-days, AQP4 expression normalized.	[103]
Mice with thrombin preconditioning subjected to ischemia (early reperfusion phase)	Early induction of AQP4 coincides with initial tissue swelling and may facilitate water clearance—limiting edema formation, although it did not prevent BBB disruption.	[104]
Rats subjected to permanent MCAO (analyzed up to 24 h)	AQP4 expression continuously increased in both the ischemic core and border regions up to 24 h, correlating with brain swelling.	[111]
Adult male rats subjected to transient MCAO (1–8 h) with 30 min reperfusion	Focal loss of AQP4 immunoreactivity in regions with high vascular permeability (indicated by fluorescein-dextran uptake) despite unchanged overall AQP4 mRNA/protein levels; minimal astrocyte death observed.	[105]
Mice subjected to 90 min MCAO followed by reperfusion (assessed at 24- and 72 h)	Significant loss of perivascular AQP4 in the striatal core at 24 h (with no recovery) and partial recovery in neocortex by 72 h; cortical border zones showed a slight increase in AQP4.	[106]
Rodent model of discrete cortical ischemia (examined one-week post-insult)	Loss of AQP4 from astrocytic end-feet, disassembly of supramolecular AQP4 complexes, and downregulation of the AQP4 ex isoform, suggesting a role in facilitating astrocyte mobility during incipient scar formation.	[112]
Mice subjected to permanent MCAO for 4 and 24 h	AQP4 immunoreactivity decreased in the striatum and varied in the cortex, delineating ischemic tissue.	[113]
Rat focal cerebral ischemia model	Cortical (grey matter) regions exhibited reduced perivascular AQP4 and ~9% swelling, while white matter showed increased AQP4 (2.2–6.2× higher) with ~40% swelling, indicating regional heterogeneity in edema formation.	[59]
*AQP4* knockout vs. wild-type mice subjected to 1 h transient MCAO with 23 h reperfusion	AQP4 deficiency resulted in a 39% reduction in infarct volume, a 23% reduction in cerebral edema, and a 31% decrease in BBB leakage, with diffusion MRI showing lesser ADC reduction around the occlusion site.	[65]
*AQP4* knockout mice vs. wild-type controls subjected to transient MCAO (3–14 days follow-up)	AQP4 deletion resulted in reduced lesion volume, decreased neuronal cell death and neuroinflammation, improved motor recovery, and lower mortality.	[66]
*AQP4* knockout mice assessed via oxygen-17 MRI and immunohistochemistry	*AQP4* deletion led to significantly reduced water exchange across the BBB and a 22% increase in cortical capillary density, suggesting an adaptive vascular response to chronic AQP4 loss.	[108]
Wild-type and α-syntrophin knockout mice (lacking perivascular AQP4) subjected to 90-min MCAO, treated with hypertonic saline for 48 h	Hypertonic saline reduced brain water content and mitigated BBB disruption in wild-type mice but had no effect in α-syntrophin knockout mice, indicating that the perivascular AQP4 pool is essential for the anti-edema effect of osmotherapy.	[107]
Rodent model of cerebral ischemia-reperfusion under hyperglycemic conditions	Hyperglycemia disrupted the continuity of perivascular AQP4 in the cortical penumbra and reduced fluorescence intensity and polarity of AQP4 in the striatal penumbra, leading to increased cellular swelling in the striatum.	[110]
Transgenic mice overexpressing ET-1 in astrocytes, subjected to transient MCAO	Overexpression of ET-1 exacerbated neurological deficits, increased infarct size, worsened BBB disruption, and elevated brain water content with enhanced AQP4 expression in astrocytic end feet.	[114]
Rat transient MCAO model and primary astrocyte cultures (evaluated at 3–7 days post-reperfusion)	Elevated AQP4 expression in peri-infarct and core regions was closely correlated with increased inflammatory markers (e.g., IL-1α); IL-1α from microglia-derived cells was shown to upregulate astrocytic AQP4, thereby exacerbating edema.	[109]

ADC, apparent diffusion coefficient; AQP, aquaporin; BBB, blood–brain barrier; ET-1, endothelin 1; IL, interleukin; MCAO, middle cerebral artery occlusion; MRI, magnetic resonance imaging.

**Table 6:** AQP4 modulation as a therapeutic strategy in experimental studies of ischemic stroke.

Molecule/Intervention	Experimental Model	Main Findings	Ref.
Atorvastatin	Rats; permanent MCAO via intraluminal suture (with sham, MCAO, and pretreatment groups)	Reduced infarct volume and brain water content, improved neurological deficits, and downregulated AQP4.	[115]
Simvastatin	Rats; MCAO model	Decreased neuronal degeneration and brain edema; lowered phospho-CaMK II and AQP4 expression.	[125]
Acetazolamide	Rats; ischemic stroke induced by bilateral carotid artery ligation	Reduced brain water content and AQP4 mRNA/protein expression, thereby alleviating cerebral edema and dysfunction.	[126]
Acetazolamide (meta-analysis)	Various animal models of ischemic stroke (systematic review/meta-analysis)	Inhibited AQP4 expression and reduced brain edema in the early stages post-stroke, though neurological benefits remain uncertain.	[117]
Treadmill pre-training	Rats; transient MCAO following 2-weeks of treadmill exercise	Downregulated AQP4, reduced brain edema, and improved BBB integrity and neurological scores.	[127]
Remote ischemic post-conditioning	Rats; transient MCAO with intermittent hindlimb occlusion	Improved neurological function, reduced infarct volume and edema, and decreased AQP4 expression in astrocytes.	[119]
Propofol (post-conditioning)	Rats; transient MCAO-induced ischemia/reperfusion injury	Reduced brain edema and BBB damage by decreasing MMP9 and AQP4 expression, leading to improved neurobehavioral outcomes.	[120]
Propofol (pre-treatment)	Rats; transient MCAO with 90-min occlusion	Attenuated cerebral edema and reduced AQP4 overexpression in the ischemic border zone; no significant change in infarct volume.	[128]
TGN-020 (AQP4 inhibitor)	Rats; non-reperfusion ischemia induced by MCAO	Reduced edema, gliosis, albumin extravasation, and apoptosis; improved overall outcome.	[129]
TGN-020 (AQP4 inhibitor)	Rats; MCAO model with MRI follow-up	Reduced brain swelling, lesion volume, and peri-infarct astrogliosis; improved functional recovery.	[116]
Methylene blue	Rats; transient MCAO with MRI evaluation	Ameliorated cytotoxic and vasogenic edema by blocking AQP4 upregulation and ERK1/2 activation.	[130]
Estradiol	In vitro cultured astrocytes exposed to ischemic factors (hypoxia, AVP, OGD)	Abolished astrocyte swelling induced by AVP/hypoxia and reduced AQP4 abundance with prolonged exposure.	[131]
Estradiol	Mice; MCAO model comparing males, females, and ovariectomized females	Preserved AQP4 levels and reduced brain edema in females; effect reversed by estrogen receptor antagonism.	[132]
LncRNA MALAT silencing (via miR-145)	In vitro OGD/reoxygenation and mouse MCAO model	Reduced AQP4 expression via miR-145 modulation, leading to decreased infarct area and neuronal injury.	[123]
LncRNA SNHG14 knockdown	Mice (MCAO) and BV2 cells under OGD	Reduced inflammation and oxidative stress, decreased AQP4 via miR-199b modulation, attenuating ischemic injury.	[133]
LncRNA MALAT1 silencing (via siRNA)	Rats (MCAO) and in vitro OGD/reoxygenation model	Decreased AQP4 expression, enhanced cell viability, and reduced apoptosis.	[122]
MicroRNA-145 overexpression	Primary cultured astrocytes under OGD	Promoted astrocyte health, reduced apoptosis, and decreased AQP4 expression by directly targeting AQP4.	[134]
MicroRNA-29b overexpression	Patients and mice with ischemic stroke; in vivo experiments	Reduced infarct volume, edema, and BBB disruption; downregulated AQP4 expression.	[124]
MicroRNA-29a overexpression	Primary cultured astrocytes subjected to OGD	Reduced LDH release, apoptosis, and AQP4 expression, protecting astrocytes against ischemic injury.	[135]
AER-270/AER-271 (functionalized phenylbenzamides)	Models of CNS injury: water intoxication and MCAO-induced ischemic stroke	Inhibited AQP4-mediated water permeability, reduced cerebral edema, and improved neurological outcomes.	[136]
Edaravone	Rats; transient focal ischemia (MCAO model)	Reduced infarct area and neurological deficits while markedly lowering AQP4 immunoreactivity.	[118]
ALDH2 activation (via Alda-1)/inhibition (via cyanamide)	Rats; MCAO-induced ischemic stroke	Alda-1 improved neurological deficits and reduced infarct size, edema, and AQP4 expression; cyanamide worsened outcomes.	[137]
AVP V1 receptor antagonist	Mice; 60-min MCAO model	Attenuated infarct volume and brain edema; modulated AQP4 expression; V2 receptor antagonist showed no benefit.	[138]
Bumetanide	Mice (WT and *α-Syn*^−/−^) subjected to 90-min MCAO with 24–48 h reperfusion	Reduced infarct volume and brain edema in WT mice, associated with decreased AQP4 expression; effect absent in *α-Syn*^−/−^ mice	[139]
Goreisan	Mice; 4 h MCAO	Decreased brain water content and AQP4 upregulation, with improved motor coordination post-stroke.	[140]
Mesenchymal stem cells (MSCs)	Mice; 90-min transient MCAO with intracranial MSC transplantation	Improved neurological scores, reduced brain edema and BBB leakage, and inhibited AQP4 upregulation	[141]
Probenecid	Mice; transient MCAO (1 h)	Reduced infarct size and brain edema, inhibited HMGB1 release, and attenuated AQP4 expression.	[142]
Normobaric Oxygen (NBO)	Rats; transient MCAO (120-min) with 48 h reperfusion	Improved neurological scores and reduced edema by decreasing AQP4 expression; 100% NBO more effective than 60%.	[121]

ALDH, aldehyde dehydrogenase; AQP, aquaporin; α-Syn, alpha-syntrophin; AVP, arginine vasopressin; BBB, blood–brain barrier; CaMK, calmodulin kinase; ERK, extracellular signal-regulated kinase; HMGB1, high mobility group box 1; LncRNA, long non-coding RNA; MCAO, middle cerebral artery occlusion; MMP, matrix metalloproteinase; MRI, magnetic resonance imaging; MSCs, mesenchymal stem cells; NBO, normobaric oxygen; OGD, oxygen-glucose deprivation; siRNA, small interfering RNA; WT, wild type.

**Table 7:** Experimental studies investigating AQP4 in experimental SAH models.

Experimental Model	Main Findings	Ref.
Murine model of SAH induced by blood injection into the basal cisterns. Wild-type and *AQP*^−/−^ mice were used.	*AQP4*-null mice exhibited greater brain edema than wild-type mice, followed by higher intracranial pressure and worse neurological deficits.	[149]
Rat SAH induced by a suture in the middle and anterior cerebral artery. Sham rats used; p53 inhibitor used.	p53 mediates cytotoxic edema following SAH via upregulation of AQP4. This expression was regulated by p38 MAPK.	[143]
SAH induced via injection of autologous blood into the cisterna magna. Sham rats used. Hydrocephalus diagnosed by histological identification.	Higher AQP4 levels in SAH-induced hydrocephalus and correlation with its severity.	[144]
Rat SAH induced via endovascular perforation of the circle of Willis. Sham rats used.	Loss of capillary coverage by AQP4-positive astrocytes’ end-feet at 4 days after SAH and astrocyte cell swelling.	[148]
Murine SAH was induced by injection of fresh unheparinized arterial blood into the cisterna magna; *AQP4*^−/−^ and wild-type mice.	After SAH, *AQP4*-null mice had a decreased blood diffusion from the perivascular space to the brain parenchyma. No neurological deficits compared with the sham group.	[150]
Perforation of the bifurcation of the anterior and middle cerebral arteries in mice inducing SAH. Sham mice used.	High AQP4 levels 6 to 72 h after SAH. Reduction of perivascular localization of AQP4. Stromal AQP4 expression was higher. PACAP treatment promoted perivascular AQP4 polarization 24 h after SAH by SUR-1 downregulation.	[145]
Murine SAH model induced by autologous blood injection into the cisterna magna.	SAH increased hippocampal AQP4 and decreased the polarization of astrocyte AQP4.	[151]
Rat SAH model induced via endovascular perforation. *AQP4* knockout rats were compared to wild-type.	*AQP4* knockout aggravated the function of the glymphatic system. The water content in the whole brain increased and the neurological deficits were more intense.	[146]
Murine SAH model induced by transfusing blood into the cisterna magna.	Increased levels of AQP4 24 h after SAH. AQP4 expression varied at different cortical sites. Depolarization was observed at all time points and correlation between AQP4 and the amount of DAC partial protein expression. AQP4 levels in the anterior cortex were significantly higher.	[147]

AQP, aquaporin; DAC, dystrophin-associated complex; MAPK, mitogen-activated protein kinase; PACAP, pituitary adenylate cyclase-activating polypeptide; SAH, subarachnoid hemorrhage; SUR-1, sulfonylurea receptor 1.

**Table 8:** AQP4 as a therapeutic target in experimental SAH.

Molecule/Intervention	Experimental Model	Main Findings	Ref.
Hypoxia-inducible factor 1α (HIF-1α)	Prechiasmatic cistern perforation SAH model in rats	Reduced brain edema via inhibition of AQP4.	[153]
Hydrogen sulfide (H_2_S)	Endovascular perforation SAH model in rats	Downregulation of AQP4; reduced edema.	[154]
Atorvastatin	Artery blood was injected into the cisterna magna in a rabbit SAH model; sham group used	Downregulation of AQP4 at 72 h; reduced edema.	[156]
Atorvastatin	Rat SAH. Endovascular perforation	Downregulation of AQP4 in a dose-dependent manner; reduced edema.	[157]
Salvinorin A	Rat SAH. Cerebral artery perforation; sham group used	Downregulation of AQP4 in the basilar artery and hippocampus.	[158]
Glutamate	Rat SAH. Cerebral artery perforation; sham group used	Glutamate elevated further AQP4 expression and edema following SAH.	[161]
Baicalin	Rat SAH. Cerebral artery perforation; sham group used	Baicalin alleviated SAH-induced early brain injury via activation of the Nrf2/HO-1 pathway and suppression of MMP9 and AQP4.	[159]
Dental pulp stem cell conditioned medium (DPSC-CM)	Rat SAH by autologous blood injection into the cisterna magna; sham group used	AQP4 downregulation; effect reversed after exposure to IGF-1.	[160]
β-hydroxybutyrate (BHB)	Murine SAH. Autologous blood injection into the cisterna magna; sham group used	Following SAH, SNTA1 levels decreased, leading to AQP4 depolarization. This action was reversed with BHB treatment.	[162]

AQP, aquaporin; BHB, β-hydroxybutyrate; DPSC-CM, dental pulp stem cell conditioned medium; HIF-1α, hypoxia inducible factor alpha; H_2_S, hydrogen sulfide; MMP, matrix metalloproteinase; SAH, subarachnoid hemorrhage.

**Table 9:** AQP4 findings in experimental ICH models.

Experimental Model	Main Findings	Ref.
Rat model of ICH by injecting quantitative collagenase into the left caudate nuclei	High perihematomal AQP4 levels at 6 h up to 1 week after ICH and correlation between AQP4 and brain water.	[163]
Mixed model; rat model of ICH by autologous blood injection and post-mortem human brains with ICH	The expression of AQP4 differs between human and rat post-ICH.	[69]
Murine ICH model induced by autologous whole blood into the striatum of *AQP4*^+/+^ and *AQP4*^−/−^ mice	AQP4 overexpression in *AQP4*^+/+^ mice. Increased edema formation, BBB disruption, and elevated neuronal death in *AQP*-null mice.	[166]
Rat model; ICH induced by infusing autologous blood into the striatum	AQP4 protein expression peaked at 5 days after ICH while mRNA peaked at 12 h; weak correlation between brain edema and AQP4 levels.	[164]
Rat model; ICH induced by infusing collagenase/heparin into the striatum	Hyperglycemia induced brain edema aggravation and significant downregulation of AQP4.	[167]
Rat model; collagenase-induced ICH; sham group used	Perihematomal AQP4 upregulation was time-dependent following ICH; AQP4 internalized to endosomes undergoing degradation into lysosomes.	[165]
Murine ICH model induced by autologous whole blood into the striatum of *AQP4*^+/+^ and *AQP4*^−/−^ mice	Increased apoptosis in *AQP4*-null mice post-ICH; higher levels of apoptosis-related proteins and worse neurologic deficits and brain edema in *AQP4* deletion.	[168]
Rat model; ICH induced by autologous whole blood into the right caudate nucleus	Downregulation of β-DG leads to depolarization of astrocyte AQP4 and worse brain edema.	[174]
Murine ICH model comparing autologous whole blood injection vs. collagen-induced ICH	BBB leakage and brain edema due to *AQP4* mRNA and MMP9 upregulation; tight junctions proteins decreased; above effects noticed in c-ICH on day 3 and on day 5 in b-ICH.	[172]
Murine ICH model induced by autologous whole blood in *AQP4*^+/+^ and *AQP4*^−/−^ mice	ROS from ICH downregulated AQP4 resulting in increased BBB permeability; *AQP4*-null mice had worse edema.	[169]
Rat model; collagenase-induced ICH	Glymphatic system blockage resulted in downregulation of AQP4, cell apoptosis, and greater brain edema.	[170]
Murine collagenase-induced ICH model in *AQP4*^+/+^ and *AQP4*^−/−^ mice	Improved glymphatic system function by AQP4 activation and hematoma reduction. Opposite effects in *AQP4*-null mice.	[173]
In vitro neurovascular unit model by co-culturing hemoglobin; circadian rhythm stimulation by short-wavelength blue-light exposure	Circadian rhythm stimulation mitigated the reduction in AQP4 expression; plausible effect in brain edema after ICH.	[171]

AQP, aquaporin; BBB, blood–brain barrier; β-DG, beta-dystroglycan; b-ICH, whole blood-induced ICH; c-ICH, collagenase-induced ICH; ICH, intracerebral hemorrhage; MMP, matrix metalloproteinase; ROS, reactive oxygen species.

**Table 10:** AQP4 as a therapeutic target in experimental ICH.

Molecule/Intervention	Experimental Model	Main Findings	Ref.
Rhubarb	Rat ICH model induced by stereospecific injection of auto-blood into caudate nucleus	Alleviated cerebral edema by reducing BBB tight junction damage and astrocyte end-feet process swelling; inhibition of transcription and translation of the *AQP4* gene.	[175]
Dexamethasone	Rat autologous blood brain injection; sham group used	*AQP4* mRNA reduced levels in perihematomal area and increased levels in brain area surrounding the third ventricle on day 3 post-ICH; brain edema reduction.	[176]
Recombinant herudin	Rat whole blood injection in caudate nucleus	Inhibition of AQP4; thrombin regulation of AQP4; decreased brain edema.	[177]
Deferoxamine	Rat ICH autologous blood injection in right caudate nucelous; healthy rats as controls	Reduced brain edema; downregulation of AQP4 by reduced iron overload.	[178]
XG-102	Murine ICH; intrastriatal collagenase injection	Increased AQP4 levels; reduced edema.	[193]
AVP V1a receptor inhibitor	Collagenase-induced ICH murine model; sham group used	Reduced AQP4 levels; reduced brain edema.	[179]
Remote ischemic post-conditioning	Collagenase-induced ICH rat model	No difference in AQP4 expression or edema.	[204]
Chinese herbs	Rat whole blood injection in caudate nucleus	Reduced brain water content and AQP4 levels.	[184]
VEGF	Murine autologous blood brain injection model; *AQP4*^+/+^ and *AQP4*^−/−^ were used	AQP4 upregulation resulted in decreased brain edema; more severe brain edema in *AQP4*^−/−^ mice.	[194]
G-CSF	Murine autologous blood brain injection model; *AQP4*^+/+^ and *AQP4*^−/−^ were used	Upregulation of perihematomal AQP4 in a VEGF-independent manner; worse edema in *AQP4* null mice; G-CSF reduce edema AQP4-dependant.	[195]
Carvacol	Murine collagenase-induced ICH; *AQP4*^+/+^ and *AQP4*^−/−^ were used	Downregulation of *AQP4* mRNA at 24 h and perihematomal protein levels in a dose-dependent manner; reduced edema.	[188]
Erythropoietin (EPO)	Murine autologous blood brain injection model; sham group used	Upregulation of perihematomal AQP4; brain edema reduction; tight junction and BBB prevention; EPO effects associated with AQP4.	[196]
Focal mild hypothermia	Rat model; thrombin-induced ICH	Downregulation of AQP4 and brain edema reduction.	[180]
Curcumin	Murine autologous blood brain injection model; sham group used	AQP4 downregulation in a dose-dependent manner; edema reduction.	[185]
Cerebrolysin	Collagenase-induced ICH rat model; sham used	Reduced edema, proinflammatory factors and AQP4 expression; upregulation of tight junction proteins.	[187]
Hyperbaric oxygen preconditioning	Autologous blood injection-induced ICH in rats; sham group used	Reduced edema and AQP4 expression in perihematomal site.	[181]
Autologous bone marrow-derived mononuclear cells (MNCs)	Rat model; ICH induced by autologous whole blood injection in left striatum	Reduced edema and AQP4 expression in perihematomal site.	[199]
Apelin-13	Collagenase-induced ICH in mice; sham used	AQP4, brain edema-associated and apoptosis-related proteins downregulation; brain edema reduction.	[186]
Adipose-derived mesenchymal stromal cells (ADSCs)	Collagenase-induced ICH in mice; sham used	Reduced edema and AQP4 expression in perihematomal site.	[201]
Propagermanium	Collagenase-induced ICH in rats; sham used	Brain edema and neurological deficits reduction; BBB integrity prevention; AQP4 downregulation.	[189]
PAR-1 inhibitor	Autologous blood injection-induced ICH in rats; sham group used	Reduced edema and *AQP4* m RNA levels in perihematomal site.	[182]
Human bone marrow mesenchymal stem cells (HBMSCs)	Rat ICH; type I collagenase and heparin brain injection	AQP4, MMP9, VEGF protein reduction; reduced edema; opposite actions and edema aggravation in the hBMSC/VEGF transfection group.	[200]
Protocatechuic acid	Collagenase-induced ICH in mice; sham used	Brain edema and BBB disruption alleviation; downregulation of AQP4 protein levels.	[190]
GHK	Collagenase-induced ICH in rats; sham used	Upregulation of miR-146a-3p and downregulation of AQP4; edema reduction.	[191]
Iron-magnetic nanoparticle-coated human umbilical-derived mesenchymal stem cells (hUC-MSCs)	Collagenase-induced ICH in rats; sham used	Dose-dependent edema reduction and AQP4 downregulation.	[202]
Butyphthalide	Rat collagenase-induced ICH model	AQP4 downregulation; BBB integrity prevention; neurological defects improvement.	[192]
Edaravone; MMP9-IN-1	Autologous blood injection-induced ICH in mice	AQP4 polarization maintenance; brain edema alleviation and BBB integrity maintenance.	[203]
GsMTx4 (Piezo1 blocker)	Murine ICH injected with autologous arterial blood into the basal ganglia	Reduced the upregulated levels of AQP4 after ICH; positive correlation of AQP4 and Piezo1.	[205]
Adjudin	Collagenase-induced ICH in mice; sham group used	Increased AQP4, tight junction and adherens junction protein levels; BBB permeability and brain cell apoptosis decreased.	[197]
Disulfiram	Collagenase-induced ICH in mice; sham group used	AQP4, MMP9, and apoptosis proteins downregulation; BBB structural proteins upregulation; edema reduction.	[183]
NETs	Rat ICH model	Aggravation of BBB integrity; tight junction proteins decreased; brain edema; increased neuronal apoptosis; perihematomal AQP4 downregulation; inhibition had opposite effects.	[198]

AQP, aquaporin; AVP, arginine vasopressin; BBB, blood–brain barrier; EPO, erythropoietin; G-CSF, granulocyte colony stimulating factor; GHK, glycine–histidine–lysine; hBMSCs, human bone marrow mesenchymal stem cells; hUC-MSCs, human umbilical-derived mesenchymal stem cells; ICH, intracerebral hemorrhage; MMP, matrix metalloproteinase; MMP9-IN-1, MMP9 inhibitor; MNCs, mononuclear cells; PAR-1, protease activated receptor-1; VEGF, vascular endothelial growth factor.

**Table 11:** Experimental studies investigating AQP2, 9, and 11 in brain injury models.

Aquaporin	Experimental Model	Main Findings	Ref.
AQP2	Cryolesion-induced TBI in mice	Increased AQP2 post-TBI; decreased AQP2 expression in *Mt1+2* knockout mice post-TBI.	[209]
AQP2	Collagenase-induced rat ICH model	ICH induced AQP2 upregulation in rat astrocytes and microglia both in vitro and in vivo. AQP2 promoted astrocyte activation and indirectly enhanced microglia transition to the M1 phenotype.	[21]
AQP2	Murine model of perioral acute inflammatory pain induced by subcutaneous injection of formalin	Increased AQP2 protein levels and altered distribution in the trigeminal ganglia.	[210]
AQP9	Closed head trauma model in rats	Elevated AQP9 mRNA and protein levels as early as 1 h post-TBI. HIF-1α inhibitor treatment reversed AQP9 upregulation.	[211]
AQP9	Modified Marmarou rat acceleration impact model	AQP9 inhibition ameliorated brain edema, neuronal damage, and improved neurobehavioural outcomes post-TBI. HIF-1α inhibition reduced both mRNA and protein levels of AQP9.	[212]
AQP9	Moderate parasagittal fluid-percussion brain injury (FPI) in rats	AQP9 mRNA and protein expression was elevated following FPI, with sustained elevation observed in both the ipsilateral parietal cortex and hippocampus.	[213]
AQP9	Severe TBI in rats	AQP9 protein and mRNA expression levels increased, reaching a maximum at 6 h post-TBI, followed by a minor reduction at 12 h.	[214]
AQP9	Modified impact/head acceleration model of diffuse TBI in rats	Increased AQP9 and HIF-1α protein levels post-TBI. Inhibition of HIF-1α reduced the TBI-induced AQP9 upregulation.	[215]
AQP9	Modified Marmarou TBI rat model (closed head trauma model)	Ethanol doses significantly decreased the elevated AQP9 mRNA and protein levels induced by TBI.	[216]
AQP9	TBI model of cold injury to the primary motor cortex in rats	AQP9 expression was reduced in agmatine-treated rats 7 days post-TBI.	[217]
AQP9	Penetrating ballistic-like brain injury (PBBI) in rats	Decreased *AQP9* mRNA levels within the first 24 h post-PBBI. AQP9 protein levels decreased at 3 days post-injury.	[74]
AQP9	Focal cerebral ischemia was induced in male B6CF1 mice by MCAO	Increased AQP9 protein expression in the infract zone, including the cortex, the ventral pallidum, and the nuclei of the amygdala on reactive astrocytes.	[218]
AQP9	Transient focal ischemia was induced in male ICR-CD1 mice by MCAO	AQP9 protein expression increased with time post-ischemia, independently from swelling in mice.	[102]
AQP9	MCAO followed by reperfusion in male Sprague Dawley rats	Inhibition of either HIF-1α or AQP9 halted the progression of edema, while increasing intracellular glycerol in rats.	[219]
AQP9	Ischemic stroke model generated by MCAO in male Sprague Dawley rats	Ethanol administration post-stroke reduced the expression of AQP9, MMP2, and MMP9, while simultaneously ameliorating brain edema and BBB leakage in rats.	[220]
AQP9	Rats with permanent MCAO	Inhibition of p38 with SB203580 prior to injury resulted in decreased levels of both AQP9 and phosphorylated p38 post-MCAO in rats.	[221]
AQP9	Global cerebral ischemia was achieved in rats by occlusion of bilateral common carotid arteries combined with hypotension for 20-min followed by reperfusion for 72 h	Pre-treatment of rats with flurbiprofen reduced *AQP9* mRNA expression in comparison to the I/R group, while demonstrating a dose-dependent effect up to 72 h post-injury.	[222]
AQP9	ICH was induced in rats by whole blood injection in the caudate nucleus	Downregulation of AQP9 expression after ICH, indicating that thrombin might play a key role in AQP9 regulation.	[177]
AQP9	ICH was induced in mice by autologous blood infusion	Curcumin suppressed elevated brain AQP9 mRNA levels and protein levels in astrocytes of ICH mice.	[185]
AQP9	Collagenase-induced ICH in mice	*AQP9*-null ICH mice demonstrate decreased neovascularization and brain cell proliferation, and greater behavioral dysfunction in comparison to wild-type ICH mice.	[223]
AQP9	Collagenase-induced ICH in male Sprague Dawley rats	Increased hippocampal AQP9 protein levels. AQP9 negatively correlated with brain angiogenesis, neuronal survival, and BBB function.	[224]
AQP9	Mannitol-induced hyperosmotic stress in rats	Increased both mRNA and protein expression levels of AQP9.	[225]
AQP11	The established cell lines for astroglia (1321N1) and neurons (SHSY5Y) were studied in response to inflammation (LPS, 10–100 ng/mL, 24 h) and hypoxia (5 min N2, followed by 0 to 24 h normoxia)	*AQP11* transcripts were upregulated in astroglia and neurons. Increased AQP11 expression reduced subsequent H_2_O_2_-induced MDA responses compared to controls.	[35]
AQP11	Collagenase-induced ICH rat model	The miR-27a-3p mimic effectively suppressed AQP11 and mitigated post-ICH complications.	[226]

AQP, aquaporin; BBB, blood–brain barrier; HIF-1α, hypoxia-inducible factor 1α; ICH, intracerebral hemorrhage; I/R, ischemia/reperfusion; FPI, fluid percussion brain injury; LPS, lipopolysaccharide; MCAO, middle cerebral artery occlusion; MDA, malondialdehyde; miR, microRNA; MMP2, matrix metalloproteinase-2; MMP9, matrix metalloproteinase-9; Mt1+2, metallothionein I and II; PBBI, penetrating ballistic-like brain injury; TBI, traumatic brain injury.

**Table 12:** AQPs as targets in the brain: perspectives and limitations.

AQP	Expression in the Brain	Injury Context	Therapeutic Potential	Limitations
AQP4	Astrocyte end-feet (BBB, glia limitans)	TBI, stroke, vasogenic/cytotoxic edema	Edema control (phase-specific); glymphatic clearance; neuroprotection	Dual role in edema; timing critical; limited drug options
AQP2	Low/induced in hypothalamus (mostly renal)	Hyponatremia/DI post-injury (SIADH, trauma)	Indirect control of systemic water balance via vasopressin axis	Not a CNS target; systemic effects only
AQP9	Astrocytes, some neurons	Stroke, epilepsy, hypoxia	Support energy metabolism (glycerol/lactate transport); cell survival	Less studied; few tools to modulate expression/function
AQP11	Low expression; intracellular (ER of glia/neuron)	Ischemia, TBI, oxidative stress	Reduce ER stress and astrocyte inflammation; experimental neuroprotection	Poorly characterized; intracellular; no specific modulators

AQP, aquaporin; BBB, blood–brain barrier; DI, diabetes insipidus; ER, endoplasmic reticulum; SIADH, syndrome of inappropriate antidiuretic hormone secretion; TBI, traumatic brain injury.
